# Species interactions shape the thermal performance curve and distribution of tropical *Drosophila* species

**DOI:** 10.1002/ecy.70451

**Published:** 2026-07-08

**Authors:** Vanessa Kellermann, Antti Miettinen, Carla M. Sgrò, Tarmo Ketola, Mads F. Schou, Belinda van Heerwaarden

**Affiliations:** ^1^ School of Agriculture, Biomedicine and Environment La Trobe University Melbourne Victoria Australia; ^2^ Department of Biology Aarhus University Aarhus Denmark; ^3^ School of Biological Sciences Monash University Melbourne Victoria Australia; ^4^ Department of Biology The University of Turku Turku Finland; ^5^ School of Bioscience The University of Melbourne Melbourne Victoria Australia

**Keywords:** competition, distributional limits, egg‐to‐adult viability, species interactions, thermal performance curve

## Abstract

Decades of research have demonstrated the importance of temperature in dictating species distributional limits. We know species interactions also play a key role, but we lack a predictive framework for understanding when and where species interactions will be more important than temperature in shaping distributional limits. In the current study, we determine how species interactions impact thermal performance for egg‐to‐adult viability (as a proxy for fitness), a commonly used method for predicting climate change vulnerability. We do so by contrasting thermal performance curves (TPCs) with and without species interactions in six species of *Drosophila* (three tropical and three subtropical species) across a range of temperatures (16–30°C). We found that thermal optimum (*T*_OPT_) was largely insensitive to species interactions, but the presence of species interactions altered the thermal sensitivities in several species and lowered the maximum performance (*P*_MAX_) compared to single‐species cultures. This suggests that competition was the dominant form of species interactions rather than facilitation. While theory proposes species interactions are more important in shaping warm‐range limits (equatorial/low elevation/low latitude) but not cool‐range limits (poleward/high elevation/high latitude), we found evidence that species interactions have larger effects at the cool range of the TPC. Moreover, species that were more sensitive to species interactions (i.e., experienced the largest effect on egg‐to‐adult viability) tended to be tropical species with restricted distributions, further supporting species interactions as an essential element shaping cool‐range distributional limits. Given that species interactions can influence thermal performance and are likely to shape species range limits, integrating species interactions into predictive models will be necessary to predict how species distributions will shift under climate change.

## INTRODUCTION

Unprecedented climate change will significantly impact species abundance, distribution, and persistence (Parmesan & Yohe, 2003). Abiotic variables, in particular temperature, are often assumed to be the most important drivers of species distributions and, consequently, responses to climate change (Addo‐Bediako et al., 2000; Kellermann, Loeschcke, et al., 2012; Kellermann, Overgaard, et al., 2012). However, temperature alone cannot explain species distributional limits, and other processes, such as species interactions, are likely involved (Alexander et al., 2022; Diamond et al., 2012). Nevertheless, predictions of species' climate change risk predominantly focus on temperature (Buckley et al., 2022; Deutsch et al., 2008; Gilman et al., 2010). The emphasis on temperature in predictions of species' climate change risk is based on compelling research showing, broadly across ectotherms, that environmental temperature is a good predictor of a species' thermal tolerance (Addo‐Bediako et al., 2000; Kellermann, Overgaard, et al., 2012). Alongside static estimates of thermal tolerance, thermal performance curves (TPCs), which capture how fitness changes across a temperature continuum (Sinclair et al., 2016), have increasingly been used for predicting distributional shifts under climate change (Deutsch et al., 2008; Kingsolver et al., 2013). These studies suggest that climate change vulnerability is likely to be highest in the tropics and subtropics (Deutsch et al., 2008; Kingsolver et al., 2013). However, we may be under or overestimating climate change vulnerability by not considering the role of species interactions in shaping current and future distributions.

Species interactions can have negative, positive, or neutral effects on fitness through competition, facilitation, or mutualism, respectively, and have long been thought to shape distributional limits (Wiens, 2011; Wisz et al., 2013). While it is challenging to measure the fitness effects of species interactions in the field, empirical laboratory studies have shown wide‐ranging impacts on fitness (Ketola et al., 2016), thermal preferences (Davis et al., 1998), and even evolutionary trajectories (Bolnick, 2001; Fiegna et al., 2015; Lawrence et al., 2012). An emerging theme from these studies is that the direction and effect of these interactions (competition, facilitation, and mutualism) can be highly dependent on the interacting species and experimental temperature (Comeault & Matute, 2021; Davis et al., 1998). In *Drosophila* species, the presence of competitor species can cause a change in their thermal preference (Davis et al., 1998), while in fish, cool‐adapted species shift their distribution to avoid competition with warm‐adapted species (Milazzo et al., 2013). As climate change reshuffles communities, creating novel interactions among species, the resilience of species to climate change will be dictated by interactions among community composition, temperature, physiology, fitness, and evolution (Gilman et al., 2010; Taniguchi & Nakano, 2000; Wethey, 2002). There is growing recognition that climate change resilience metrics like the TPC fail to capture ecological realism (Sinclair et al., 2016), yet empirical data explicitly testing how competition influences the TPC are lacking (Sunday et al., 2024).

One theory that could help us predict the role of species interactions in shaping distributional limits is the cool/warm range edge/limit hypothesis (Darwin, 1859; Dobzhansky, 1950). This theory predicts that biotic interactions are more critical in shaping warm‐range limits (i.e., range limits at warmer temperatures, lower latitudes, and elevations), while abiotic variables shape cool‐range limits (i.e., limits at cooler temperatures, higher latitudes and elevations) (Paquette & Hargreaves, 2021; Schemske et al., 2009). Species diversity increases from temperate to tropical environments (latitudinal species diversity gradient), and this diversity gradient drives differences in the relative impact of species interactions across latitudes (and hence environments) (Darwin, 1859). According to this range limit theory, distributional limits arise at the warm‐range limit of a species because of increasing negative interactions (i.e., competition) or because facilitative interactions begin to decline (Paquette & Hargreaves, 2021). At the same time, abiotic conditions tend to be less variable at the warm‐range limit, increasing the importance of species interactions (Dobzhansky, 1950). At the cool‐range limit, biotic interactions decline, and abiotic variables are more volatile, making them more important in driving distributional limits than biotic interactions (Schemske et al., 2009). However, the support for the species interactions range limit theory is mixed, with studies finding some support (Paquette & Hargreaves, 2021; Schemske et al., 2009), but others finding little evidence supporting the increased importance of biotic interactions at the warm‐range limit (Cahill et al., 2014). Studies on range limits tend to focus on abiotic causes of range limits, with few studies testing both abiotic and biotic factors in a single study (Cahill et al., 2014; Paquette & Hargreaves, 2021; but see O'Brien et al., 2018).

The current study examines how species interactions impact the TPC of six species of vinegar flies (*Drosophila*) originating from low to intermediate latitudes. While species interactions can occur between taxa at different trophic levels (i.e., predation and parasitism), we explore *Drosophila*/*Drosophila* interactions because many *Drosophila* species coexist and share resources in a given environment (Atkinson & Shorrocks, 1984; McKenzie & McKechnie, 1979; Ribeiro et al., 2023). *Drosophila* species are likely to experience exploitative competition at the larval development stage as they compete for a finite resource (Krijger et al., 2001), as well as interference competition where the presence of interacting species affects traits even at low densities (Ayala, 1970). We focused on egg‐to‐adult viability because this trait is likely influenced by competition and because egg‐to‐adult viability is a fitness‐related trait likely to shape population persistence (Kristensen et al., 2015; Magiafoglou et al., 2002).

To examine *Drosophila*/*Drosophila* species interactions, we initiated one‐, two‐, and three‐species cultures across a range of temperatures. We then leveraged the thermal performance framework to test (1) how species interactions influence egg‐to‐adult survival/viability, as a proxy for fitness, whether that be competition or facilitation, (2) whether developmental temperatures influence the outcomes of species interactions, and (3) whether species interactions alter the thermal optimum (*T*_OPT_) and maximum performance (*P*_MAX_), which are common predictors of vulnerability to climate change (Deutsch et al., 2008; Sinclair et al., 2016). Because we initiated two‐ and three‐species cultures, we also have the capacity to ask (4) whether indirect effects due to the presence of a third species change the pairwise interactions or influence thermal performance. We then ask (5) if the impact of species interactions on each species' TPC is associated with their cool‐range limit as previously predicted (Paquette & Hargreaves, 2021). If species interactions have disproportionately large effects on species occupying different latitudes, this could have implications for species' vulnerability under climate change.

## MATERIALS AND METHODS

### Source populations

**FIGURE 1:**
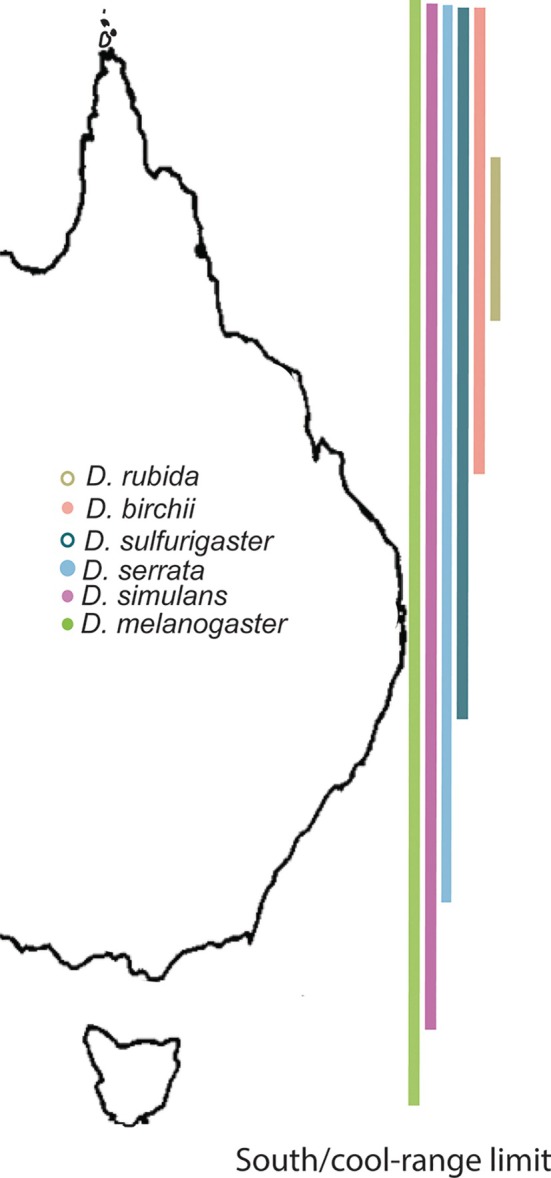
The distribution of each of the six *Drosophila* species in Australia. Colored dots reflect the collection point for each species (Appendix S1: Table S1 for exact GPS locations); the solid‐colored lines represent the known distribution of each species.

We chose six species of *Drosophila* found in Australia at either low or intermediate latitudes that can loosely be defined as tropical versus subtropical species (Figure 1). Species occupying environments between 0 and 23° latitude, with annual precipitation >1500 mm per year and annual mean temperatures >18°C: *D. birchii* (bir), *D. sulfurigaster* (sulf), and *D. rubida* (rub) were deemed tropical species, while those occupying higher latitude environments (>23° latitude) were considered sub‐tropical species: *D. melanogaster* (mel), *D. serrata* (ser), and *D. simulans* (sim). In the tropical group, bir is endemic to Australia, while sulf and rub are considered widespread across tropical South‐East Asia (Bock, 1976). In the subtropical group, mel and sim are found globally, while ser is endemic to Australia with a latitude range limit closer to the equator (Bock, 1976). Species within each tropical/subtropical group have overlapping distributions and are often found at the same collection sites (Figure 1), suggesting these species are likely competing for resources and oviposition sites (Kellermann, unpublished data).

The species were collected from the field between 2012 and 2014 via sweep netting over rotten bananas (experiments were completed following 15–36 generations in the lab as follows: Appendix S1: Table S1). The tropical species (bir/rub/sulf) were collected from rainforest sites. The subtropical species mel/sim were collected from a household compost bin, while ser was collected from native vegetation within a regional town. A mass‐bred population was initiated for each species using F2 offspring from 20 to 25 lines derived from wild‐caught females (iso‐female lines) (Appendix S1: Table S1). Mass‐bred populations of each species were maintained at a population size of approximately ~750 on yeast potato media (Kutz et al., 2019) at 25°C with a 12:12‐h light cycle. Before the experiment, to reduce carry‐over density‐dependent effects during developmental stages, we restricted the amount of time for egg laying, such that vials (5‐mL media) produced fewer than 60 emerging adults (see Appendix S1: Figure S1 for experiments showing density ~80 impacts egg‐to‐adult viability).

### Experimental setup

#### Effect of interspecific interactions on egg‐to‐adult viability

To examine how species interactions shape the TPC, flies were developed from egg to adult with and without species interactions under five constant temperatures: 16, 20, 23, 28, and 30°C. We selected these developmental temperatures to capture the general shape of the thermal performance—specifically the decline in egg‐to‐adult viability at warm and cool temperatures, as well as the thermal optima (*T*_OPT_). Based on previous work, we expected tropical species would not survive 30°C (Kellermann & Sgrò, 2018) (but widespread species would) and that the *T*_OPT_ would be between 20 and 23°C (Kellermann et al., 2019). Because we were interested in whether more complex communities would influence the outcomes of species interactions, we initiated treatments with two‐ and three‐species cultures, and single‐species cultures separately within each tropical/subtropical group. For the two‐species cultures, all possible combinations of interactions were set up within the habitat groups, resulting in six different species interactions initiated across all species, replicated across all developmental temperatures (tropical: bir vs. sulf, bir vs. rub, and sulf vs. rub; subtropical: mel vs. sim, mel vs. ser, and ser vs. sim). For the three‐species cultures, we set up all tropical species in one culture and all subtropical species in another culture. Due to the size of the experiment, the two‐ and three‐species cultures were set up at different times, each with their own set of single‐species cultures (control).

Prior to the experiment, 50–100 females from each species were placed onto six laying pots (250‐mL plastic containers with a hole cut out of the top to allow for flies to be moved in and out of the container, sealed with a styrofoam plug) containing standard fly food (as above) with the addition of double agar to encourage eggs to be laid on the surface (egg‐laying food is placed into the lid, which allows for food to be replaced by changing the lid). Live yeast was spread on the surface of the food to stimulate oviposition (Markow & O'Grady, 2006). Using a fine metal tool flattened at the end, eggs were picked from the laying lids and placed into vials containing 5 mL of standard fly food. Single‐, two‐, and three‐species cultures were initiated such that the total density of eggs (78–80 eggs) in a vial remained unchanged regardless of whether the vial contained one, two, or three species. Thus, for single‐species cultures, 80 eggs of the focal species were picked into one vial; for the two‐species cultures, 40 eggs (total = 80) of each species were placed into one vial; and for the three‐species cultures, 26 eggs (total = 78) of each species were placed into the same vial. This meant that total density was controlled rather than the level of intraspecific interactions. We chose 80 individuals based on prior intraspecific experiments that showed density effects on egg‐adult viability with 80 eggs (Appendix S1: Figure S1). For each developmental temperature and each single, two‐, and three‐species culture, we aimed to initiate 12 replicates, but for some treatments, we could only get enough eggs to set up 10 replicates. Due to the size of the experiment and the need to collect 9000–9600 eggs per species, eggs were picked over multiple days. All three‐way control/competition vials and two‐way tropical control/competition vials were picked over 2 days, while two‐way temperate control/competition vials were picked over 3 days. Vials were blocked across days so that each temperature and treatment was evenly represented. The number of eclosing individuals was scored by species. Vials were kept for several days after the last adult emerged to ensure that all adults had emerged and were included in our viability estimates. For the vials containing more than one species, flies were knocked out using CO_2_ anesthesia, and species were identified using a dissecting microscope at 4–6× magnification (Leica M80 stereo microscope [Leica, Heerbrugg, Switzerland]). Species were identified by VK primarily based on experience, using diagnostic features including different combinations of body color, banding patterns, sex combs, and male genitalia (Bock, 1976). We acknowledge that the females of the species pair of mel and sim are challenging to separate with certainty at warmer temperatures. These species were distinguished by comparing the female banding patterns of the last two abdominal segments, which become lighter at warmer temperatures. Identification was 100% certain at temperatures ≤23°C and 92.50% at ≥28°C (see Appendix S1). The resulting TPCs for mel and sim should therefore be interpreted with this in mind.

### Analysis

#### Effect of interspecific interactions on the TPC for egg‐to‐adult viability

We constructed one generalized linear mixed model for each species to evaluate whether species interactions influenced TPCs. This was done in a Bayesian framework implemented in the package MCMCglmm v.2.26 in R v.3.5.1 (Hadfield, 2010). We modeled the egg‐to‐adult viability with a binomial error distribution and logit link function, which in MCMCglmm effectively deals with the potential issues of overdispersion (Hadfield, 2026) while modeling the zero‐boundary of the TPC at thermal extremes. The response variable was modeled as a binomial vector: successes (number of flies that emerged from their pupae) and failures (number of flies that did not emerge from their pupae). Each model included culture treatment as a fixed effect with five levels: for example, mel ([focal species control], mel/sim, mel/ser) and the two different three‐species culture treatments (mel [focal species control], mel/ser/sim). We also included linear and quadratic terms for temperature and their interactions with the five‐level culture treatment factor, so that a TPC was estimated for each culture treatment. This was done by computing *z*‐transformed values of temperature (temperature_*z*: mean‐centered and scaled to unit variance) before running the models with temperature_*z* and temperature_*z* ^2^. Because of the lack of independence among vials of flies initiated on the same day, we included day as a random effect.

We considered a fixed effect significant when the 95% credible interval (95CrI = the parameter has a posterior probability of 95% to lie in this interval) did not overlap with 0 and the Markov chain Monte Carlo p‐value (pMCMC) was less than 0.05 (pMCMC = proportion of iterations above or below a test value correcting for the finite sample size of posterior samples). The 95CrI is, in addition to indicating statistical significance, also used to infer the likely range of the parameter estimate, while the pMCMC can be interpreted in a similar way to *p* values obtained from an ANOVA. We suppressed the global intercept for the five‐level factor of culture treatment in all models, so that the absolute parameter estimates and 95CrI for all culture treatments can be estimated. This also enabled us to perform pairwise comparisons among the five different culture treatments within a model. Differences between culture treatments were estimated by subtracting the posterior samples from one culture treatment from the other culture treatment and calculating the posterior mode, 95CrI, and pMCMC. This was also done between the quadratic terms of temperature in pairs of culture treatments, and between the linear terms of temperature in pairs of culture treatments. In this way, we evaluated whether any two culture treatments differed in their TPC. We were particularly interested in two contrasts within each model: (1) two‐species versus focal: if the TPC of two‐species culture treatments (e.g., mel/sim) differed from the culture treatment with the relevant focal species alone (e.g., mel); (2) three‐species versus focal: if the TPC of three‐species culture treatment (mel/ser/sim) differed from the culture treatment with the relevant focal species alone (e.g., mel). To test whether the two‐species culture treatments differed from the three‐species culture treatments, we also subtracted the posteriors of the two‐species versus focal contrast from those of the three‐species versus focal contrast. This was necessary as the relevant focal culture differed between the two‐ and three‐species cultures due to the experimental blocking (Appendix S1: Table S2).

Using the posterior probabilities of the fixed effects, we calculated posterior probabilities of the *T*_OPT_ and *P*_MAX_ for each of the five culture treatments in the models. *T*_OPT_ was estimated as the vertex of the TPC (*T*_OPT_ = −*b*/2*a*), where *a* is the posterior probabilities for the quadratic term of temperature, and b is the posterior probabilities for the linear term of temperature. *P*_MAX_ was estimated by using the quadratic equation (*y* = *ax* ^2^ + *bx* + *c*), where *c* is the posterior probability of the culture treatment, to estimate *y* (egg‐to‐adult viability, *P*_MAX_) when *x* = *T*_OPT_. For models with *D. melanogaster* as the focal species, we observed strong outliers in the posterior probability of *T*_OPT_, which distorted estimates of both mode and mean. Instead, we report the median of the posterior for *T*_OPT_ and *P*_MAX_ for all species, as this limits the influence of outliers and as it was recently shown to be an unbiased estimator (Pick et al., 2023). The temperature range used to examine egg‐to‐adult viability was not broad enough or sufficiently resolved to estimate other common descriptors of the TPC (e.g., CT_MIN_, CT_MAX_), particularly for the subtropical species, so they were not estimated.

For the priors of both residual and random terms, we used the weakly informative inverse‐Gamma distribution (scale = 0.001, shape = 0.001, i.e., *V* = 1, nu = 0.002). Each model was run for 1,100,000 iterations, of which the initial 100,000 were discarded, and only every 500th iteration was used for estimating posterior probabilities. The number of iterations was based on inspection of autocorrelation among posterior samples in preliminary runs. Convergence of the estimates was checked by running the model three times and inspecting the overlap of estimates in trace plots, to ensure that levels of autocorrelation among posterior samples were low, testing with Gelman and Rubin's convergence diagnostic (potential scale reduction factors <1.1). Visual inspection of the predicted TPC, and the observed data showed that the combination of a quadratic curve and the zero‐boundary enforced by the binomial regression resulted in a good fit for most culture treatments and species. However, for ser, sulf, and sim, the model did not appropriately model the egg‐to‐adult viability at 28°C. This could potentially lead to spurious differences in the TPC between different culture treatments. Such a lack of fit at individual temperatures could potentially have been avoided using nonlinear models. However, we believe these were unfitting for our experimental design, where only five independent developmental temperatures were used, which would risk putting too much weight on the egg‐to‐adult viability at single developmental temperatures (overfitting). Instead, we tested the robustness of our results by repeating the analysis for ser, sulf, and sim while omitting the 16°C developmental temperature. This approach allowed us to model more flexibility in the curvature at high developmental temperatures. While the exclusion of 16°C improved the fit of the model at higher developmental temperatures, the results for *T*_OPT_ and culture treatment by temperature interactions did not change (Appendix S1: Figure S2 and Tables S3–S5).

#### What shapes cool‐range limits?

Under the warm/cool‐range limits hypothesis, temperature should be more important in shaping cool‐range limits at higher latitudes than warm‐range limits at lower latitudes, while species interactions should be more important in shaping warm‐range than cool‐range limits. We tested this hypothesis by asking whether the presence of other species (two‐/three‐species culture treatments) influences egg‐to‐adult viability in comparison to the single‐species (control) culture treatments by estimating the competition index CompIndex as the difference between the two‐/three‐species culture treatments and controls. To obtain these values, we randomly sampled trait values from the two‐ or three‐species and the single‐species culture treatment, respectively (Alton & Kellermann, 2023). Specifically, we calculated the proportional egg‐to‐adult viability for each vial and then calculated the mean CompIndex ± 95% CI at each developmental temperature by randomly sampling the single and two or three species culture treatment with replacement in R performing 10,000 bootstraps using the boot function. For each species, within each developmental temperature we have three CompIndex's, two for each of the two‐species culture treatments, for example, for *D. melanogaster*: mel/sim and mel/ser (note: the first species denotes the focal species) and one for the three‐species culture treatments. Our main question was whether the effect of species interactions on egg‐to‐adult viability, as a proxy for fitness, was associated with cool‐range limits. Species interactions, however, can have both positive and negative effects on fitness (competition vs. facilitation), and this can be highly dependent on the interacting species identity (Wallace, 1974); we therefore first determined whether there was directionality in the relationship between species interactions and range limits by analyzing both positive and negative values of the CompIndex. We then asked whether there was a relationship between species range limits and the sensitivity of each species to the presence of species interactions (be that competition or facilitation) by analyzing the absolute values of the CompIndex (making all values positive).

From a dataset containing known collection locations of each of the six species, we found the collection site with the lowest (warm) and highest (cool) latitude (Kellermann et al., 2020). While our initial intention was to link both cool and warm‐range limits to the CompIndex, it became clear that for the *Drosophila* species examined in this study, there was no true warm‐range limit, that is, both the tropical and widespread species are found in the tropical zone around the equator (Figure 1). For this reason, we focused on the cool‐range limit. To test for a relationship between the CompIndex and the cool‐range limit, we used mixed effects models in the lme4 package (Bates et al., 2014) in R. The full models had the CompIndex as a Gaussian response variable, with latitude of the cool‐range limit (continuous), experimental developmental temperature (continuous), and their interaction as fixed effects. Statistical significance of fixed effects was assessed using Wald *t*‐tests based on the model‐estimated coefficients. Degrees of freedom and *p* values were obtained using the Satterthwaite approximation implemented in the lmerTest package (Kuznetsova et al., 2017). Variance of fixed and random effects was partitioned using the MuMIn package (Barton, 2023). Significance testing was conducted using an analysis of variance (Type III) in R. To further dissect the significant development temperature × latitude interaction detected in the models of absolute CompIndex (see *Results*), we split the data by development temperature and tested for a relationship between CompIndex and latitude.

#### The relationship between wing size and competition

To determine whether the competitive success of the different species was associated with wing size, a commonly used proxy for body size in *Drosophila* that is correlated with thorax length (Gilchrist & Partridge, 1999; Reeve & Robertson, 1953), we removed the right wing of each species and mounted this onto a slide with double‐sided tape. We photographed 20 wings for each species at each developmental temperature using a Wild M3 dissector microscope (Leica, Heerbrugg, Switzerland) attached to a digital camera. Images were landmarked using TPSDIG (Rohlf, 2008) version 2.12 at eight longitudinal veins with the wing margin or cross veins to obtain an estimate of centroid size, estimated from the mean distance between each landmark and the mean *x* and *y* coordinates of a wing (Rohlf, 2001). Taking the CompIndex for each species at each developmental temperature (three estimates per species/per developmental temperature) and the average wing size for each species at each developmental temperature (estimated on control flies), we performed a mixed effects linear regression in R to look for a relationship between CompIndex (*Y*) and wing size (*X*). Developmental temperature was deemed a fixed effect, and species a random effect. We removed one outlier treatment bir/rub at 20°C.

#### The importance of intraspecific competition on egg‐to‐adult viability

In the current study, we focused on keeping the absolute density of flies the same across our one‐, two‐, and three‐species culture treatments. This means our treatments were exposed to different levels of intraspecific competition. We conducted preliminary experiments with two purposes. The first was to determine the number of eggs that induced an effect of species interactions on egg‐to‐adult viability. And the second was to examine the egg‐to‐adult viability of all species at five different intraspecific densities (10, 20, 40, 80, and 160 individuals) at two temperatures (23 and 26°C for tropical species and 23 and 28°C for subtropical species). Note: we chose 26°C for tropical species as we knew from previous experience that egg‐adult‐viability is extremely low at 28°C. We found that increasing intraspecific interactions had only a minor effect on egg‐to‐adult viability, and therefore, different levels of intraspecific competition between our treatments are unlikely to influence our results. For further details of the methods and results from this preliminary study, see Appendix S1: Figure S1, Supplementary methods and results. See Kellermann et al. (2026) for data and R code.

## RESULTS

**FIGURE 2:**
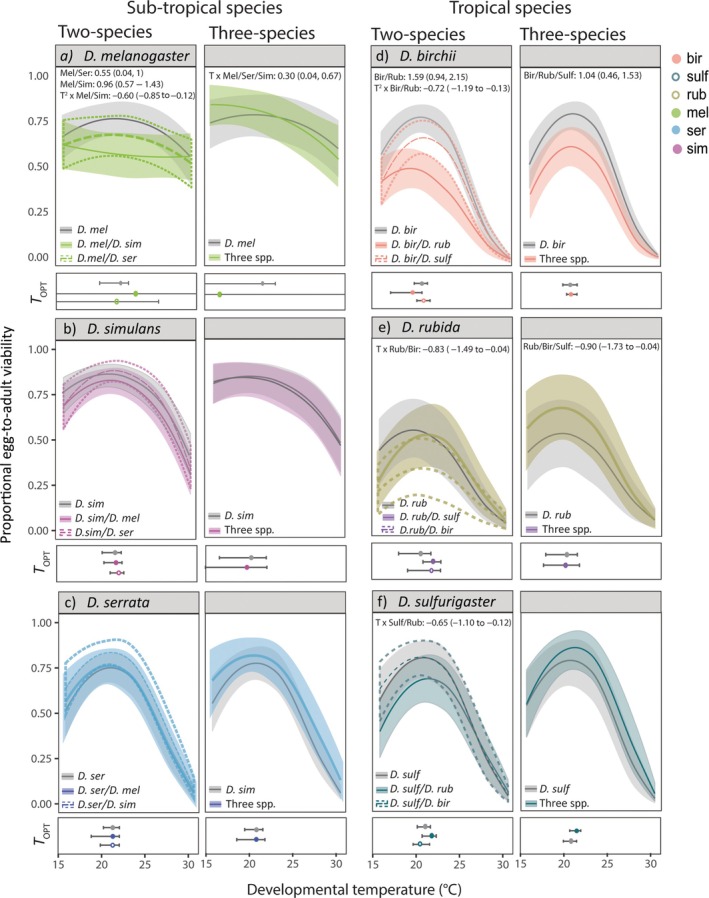
Thermal performance curves (TPCs) for egg‐to‐adult viability (proportion viable) for single (gray lines) and two or three species culture treatments (colored lines) for both subtropical (left two panels) and tropical species (right two panels), including estimates of the thermal optima (*T*_OPT_) and its 95% CrI shown below each panel. Single‐species controls were set up for the two‐ and three‐species interactions because they were set up at different times. Significant model terms from the contrast between two‐/three‐species curves to the single‐species curve are displayed above the curves (for full model outputs, see Appendix S1: Tables S6–S11; for raw data figures, see Appendix S1: Figures S3 and S4). Fitted lines and 95% credible intervals were estimated from the MCMCglmm models (Appendix S1: Tables S6–S11).

Developmental temperature had a larger effect on egg‐to‐adult viability than both two and three‐way species interactions, with egg‐to‐adult viability showing a typical bell‐shaped TPC with lower egg‐to‐adult viability at cooler and warmer temperatures (Figure 2; Appendix S1: Figures S3 and S4, Tables S6–S11). The only exception was the mel/sim culture treatment (as outlined in the methods, mel/sim = *D. melanogaster* is the focal species in the species interaction between *D. melanogaster* vs. *D. simulans*), where neither the linear nor the quadratic effect of temperature was significant (Figure 2a, mel/sim temperature: −0.10 [95% CrI, −0.32, 0.08], mel/sim temperature^2^: 0.07 [−0.20, 0.33]).

We found no evidence that the effect of the species interactions (via the Competitive Index, CompIndex) was associated with the wing size of the interacting species (Appendix S1: Figure S5).

### Do species interactions have a facilitative, competitive or mutualistic effect on egg‐to‐adult viability?

**FIGURE 3:**
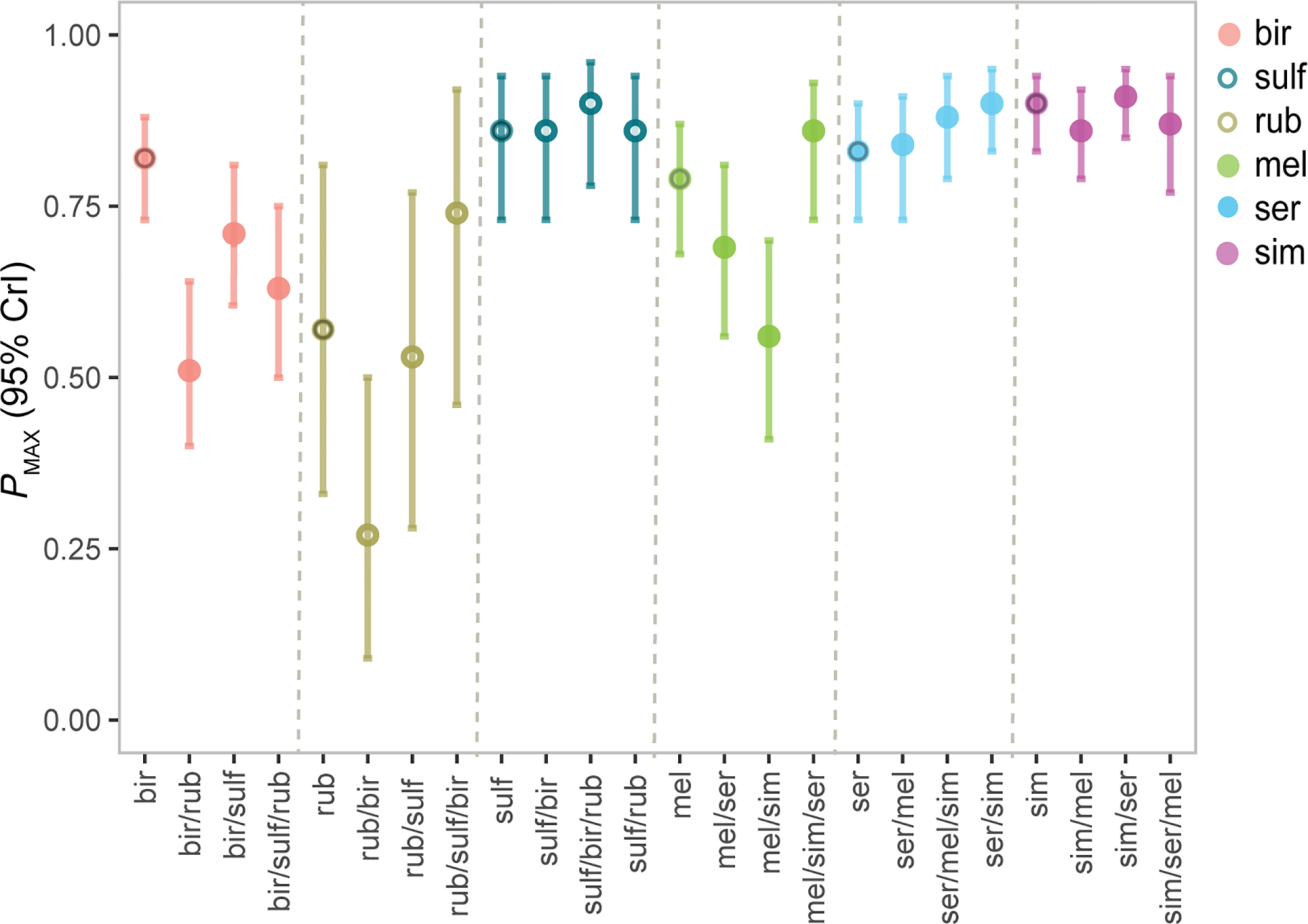
Maximum performance (*P*_MAX_) estimated from the thermal performance curve (TPC). *P*_MAX_ and the 95% CrI are shown for each culture treatment.

The presence of interacting species impacted the height of the TPC for egg‐to‐adult viability (*P*_MAX_) in a highly species‐specific manner (Figure 3; Appendix S1: Table S12). When a difference in egg‐to‐adult viability was observed between the single‐species and the two‐/three‐species culture treatment, it typically reflected a negative effect on egg‐to‐adult viability of the focal species, which we interpreted as competition among species. Two‐species culture treatments, relative to single‐species culture treatments, significantly decreased the height of the TPC for *D. melanogaster* (both mel/ser and mel/sim) (Figures 2a and 3), *D. birchii* (bir/rub) (Figures 2d and 3), and *D. rubida* (rub/bir) (Figures 2e and 3) (Appendix S1: Table S12). Three‐species culture treatments had a smaller overall impact on egg‐to‐adult viability, with declines in *P*_MAX_ (competitive effects) evident for *D. birchii* (Figures 2d and 3) and increases in *P*_MAX_ (facilitative effects) observed for *D. rubida* (Figures 2e and 3; Appendix S1: Table S12). For *D. simulans* and *D. serrata*, two‐ and three‐species culture treatments had little impact on egg‐to‐adult viability and hence *P*_MAX_ (Figures 2b,c and 3; Appendix S1: Table S12).

### Is there an interaction between temperature and species interactions?

#### Thermal optima

The *T*_OPT_ was highly conserved regardless of whether species were reared in two‐ or three‐species culture treatments versus single‐species culture treatments (see insets below the main panels in Figure 2). *T*_OPT_ were also comparable across species types (subtropical vs. tropical). For subtropical species, *T*_OPT_ ranged from 20 to 24°C, while for tropical species, *T*_OPT_ ranged from 19 to 21°C (Appendix S1: Table S13).

#### Does the temperature of rearing affect the outcome of species interactions?

If the effect of two/three‐culture treatments on egg‐to‐adult viability depended on temperature, we would anticipate the shape of the TPC to change compared to the single‐species culture treatments and drive a significant interaction between temperature and species. We found evidence for such interactions, but these effects were highly species‐specific (Figure 2; Appendix S1: Tables S6–S11). An interaction between temperature and the two‐species culture treatment was found for *D. melanogaster* (mel/sim) (Figure 2a), *D. birchii* (bir/rub) (Figure 2d), *D. rubida* (rub/bir) (Figure 2e) and *D. sulfurigaster* (sulf/rub) (Figure 2f), indicating that the presence of another species influenced the shape of their egg‐to‐adult viability TPC. For the mel/sim culture, reduced egg‐to‐adult viability in the two‐species culture relative to the single‐species culture treatment (competition) was greatest at the intermediate temperatures (Figure 2a). For all other species, the effect of competition tended to be greatest at 16–23°C, with small to negligible differences between single versus two‐species culture treatments at warmer temperatures. The bir/rub culture treatment was the only culture treatment where we saw reciprocal effects for each species; there was no evidence of one species suppressing another, but instead, the presence of each species negatively influenced the egg‐to‐adult viability of the other species (Figure 2d,e). For the three‐species culture treatments, we found significant temperature × species interactions only for *D. melanogaster*, where competition had a positive effect on egg‐to‐adult viability at the cooler temperatures, while the reverse was true at warmer temperatures (Figure 2a). We found no temperature × species interactions in any of the tropical species (Figure 2d–f), which contrasts with the two‐species culture treatments, where we found a significant temperature‐by‐species interaction in at least one of the two‐species culture treatments.

We observed indirect effects of species interactions for *D. melanogaster* and *D. rubida* in our comparisons of the two‐ and three‐species culture treatments. When we contrasted the TPC of the two‐species to the three‐species culture treatments (mel/sim, mel/ser vs. mel/ser/sim) in *D. melanogaster*, we found a significant difference between the linear component of the two‐species: mel/sim versus three‐species culture treatment (0.55 CrI: 0.08, 0.093; Appendix S1: Table S6). That is, egg‐to‐adult viability was higher in the three‐species culture than in the mel/sim two‐species culture treatment (Figure 3). These results suggest that *D. serrata*, in conjunction with *D. simulans* (mel/ser/sim), impacted the egg‐to‐adult viability of *D. melanogaster*. The same was true for *D. rubida* when contrasting the two‐species culture treatments of rub/bir with the three‐species culture treatments (rub/bir/sulf), where *D. sulfurigaster* increased egg‐to‐adult viability of *D. rubida* when in the presence of *D. birchii* (1.01 CrI: 0.16–2.16, Figure 2e).

### The role of species interactions versus climate in shaping species distributions

**FIGURE 4:**
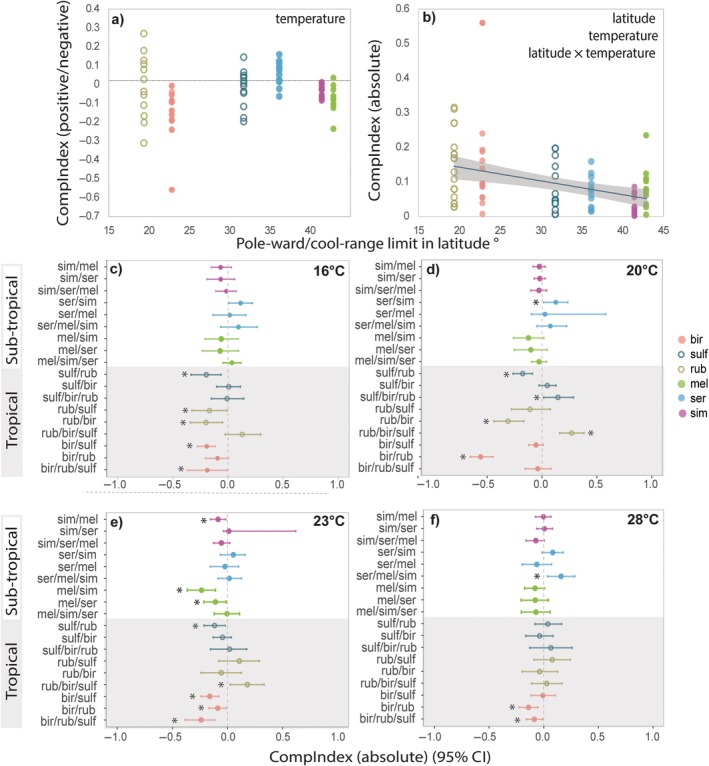
The role of species interactions versus climate in shaping species distributions. The relationship between a species' cool (poleward) latitude range limit and the species interaction index (CompIndex) using positive/negative (a) and absolute (b) values. Each dot represents the CompIndex for a given species at each developmental temperature (16/20/23/28°C) and culture treatment. Significant model terms are given, and a significant linear relationship between the CompIndex and latitude is shown with a line of best fit and the respective 95% CI around the mean prediction. (c–f) The CompIndex and 95% CI for a given species and culture treatment at each developmental temperature. * Indicates the CompIndex's with 95% CI's that do not include zero. The 30°C development temperature was not included because viability was 0 for all species except *Drosophila melanogaster* and *Drosophila simulans*.

There was no evidence to suggest that species interactions (as summarized by positive/negative CompIndex) were more important in shaping species distributions than temperature (Figure 4a; Appendix S1: Table S14). Using absolute values, we found an association between the CompIndex and latitude (cool‐range margin) (Figure 4b; Appendix S1: Table S15). This suggests that species with wider ranges (those with a range margin closer to the poles, that is, cold/high‐latitude, given that all species had very similar equatorial/warm/low‐latitude range margins) tend to be less sensitive to species interactions than species with narrower ranges. We also found, in the tropical species, that the presence of interacting species had a larger effect on egg‐to‐adult viability at cooler rather than warmer temperatures (Figure 4c–f), such that the CompIndex was significant for more treatments at 16°C (5 of 9 culture treatments) than 28°C (2 of 9 culture treatments) This trend was also apparent in our TPC analysis (Figure 2).

## DISCUSSION

Species interactions are likely a key force shaping species distributions and range limits, yet we lack a framework for predicting when species interactions will be more important than temperature. If the nature of species interactions can change across temperature, for example, from facilitation to competition, then this will likely impact species distributional shifts under climate change beyond that of temperature effects alone. Here, we tested how species interactions and temperature interact to shape egg‐to‐adult viability, a proxy for fitness, in subtropical and tropical *Drosophila* species. While *T*_OPT_ was largely insensitive to the presence of two/three species, the height of the performance curve (*P*_MAX_) was reduced by competition. The shape of the TPC was also impacted by the presence of other species, particularly at cooler temperatures, which was linked to the cool‐range limits of tropical species, suggesting a role for species interactions in shaping cool‐range limits.

The presence of other species did not shift the *T*_OPT_ of any species (Figure 2). This result is consistent with studies in *Drosophila* showing that *T*_OPT_ does not vary considerably when estimated across populations or even across studies (Kellermann et al., 2019; Klepsatel et al., 2013). However, a highly conserved *T*_OPT_ for egg‐to‐adult viability contrasts with studies showing that thermal preference (sometimes used as a proxy for *T*_OPT_) can change with the addition of interacting species (Davis et al., 1998). The change in thermal preference in the Davis et al. (1998) study occurred in the temperate species, *Drosophila subobscura*, which was outcompeted at warmer temperatures. The outcomes of species interactions are often species‐specific, and the contrasting results likely reflect the different combinations of species examined in the current study (Comeault & Matute, 2021). A large body size could also be important for the competitive success of each species, but we found no evidence that body size dictated competitive outcomes in the current study. Determining whether other traits commonly used to estimate the TPC (e.g., in *Drosophila*: fecundity, development time) are equally insensitive to species interactions remains to be tested. Nevertheless, we detected differences in the shape of the thermal performance, which could mean *T*_OPT_ is not an informative metric for estimating climate change resilience (Deutsch et al., 2008). Studies have also shown that estimates of *T*_OPT_ can generally be quite conserved across traits in *D. melanogaster* (Kellermann et al., 2019).

While we did not find that multispecies culture treatments influenced *T*_OPT_, we did find evidence for competitive effects on mean egg‐to‐adult viability (*P*_MAX_), but these effects were species‐specific (Figure 3). The context‐dependent nature of species interactions has been demonstrated before (Comeault & Matute, 2021; Davis et al., 1998), making it challenging to develop a predictive framework for when species interactions will influence fitness. It is possible that the idiosyncratic nature of species interactions could depend on the coevolution of these interactions in the wild, that is, species with a long history of species interactions are more or less likely to show facilitation or competition. It is difficult to explicitly test this here, given that we know very little about the ecology of *Drosophila* beyond the fact that their distributions and collection sites overlap. It is noteworthy, however, that competition was the dominant form of species interactions, meaning that at least in the species studied here, the presence of other species tended to have a negative effect on egg‐to‐adult viability.

Most competition studies examine pairwise competitive interactions and assume competitive effects to be hierarchical (Soliveres & Allan, 2018). Under a hierarchical model, the dominant species will exclude all others, the second dominant will exclude all but the dominant, and so forth. However, this ignores the possibility of indirect effects of a third or more species. That is, the presence of additional species changes the competitive effect of pairwise interactions, and no species is the “alpha” competitor; instead, the effects of competition depend on the composition of species (intransitive interactions) (Soliveres & Allan, 2018). These indirect effects of competition could both promote and impede coexistence, but stable coexistence can emerge when a third species reduces competitive effects between two species (Levine et al., 2017). Such intransitive interactions could include the “rock‐paper‐scissors” cyclic dominance observed in microbial (Kerr et al., 2002) and vertebrate systems (Sinervo & Lively, 1996). In the current study, there was evidence for indirect effects occurring in our three‐species culture treatments, such that the effects of competition were generally smaller in the three‐species than in the two‐species culture treatments. In some cases, competitive effects evident in some pairwise interactions were reversed when an additional species was included, indicating that direct and indirect effects of competition can influence TPC estimates. In communities with large numbers of species, it could also be that only a few “key species” dictate interactions, with negligible effects on others (Ketola et al., 2016). While most studies focus on pairwise impacts of competition, there is some evidence for indirect effects occurring in *Drosophila* (Budnik et al., 1997; Worthen & Moore, 1991). Still, evidence for between‐species indirect effects is rare (Levine et al., 2017).

The temperature dependence of species interactions has been shown in several species and could have significant implications for species' future vulnerability if interactions shift from positive (facilitative) to negative (competitive) under climate change (Comeault & Matute, 2021; Gilman et al., 2010). We did see some evidence of a change in interaction type in the *D. melanogaster* three‐species treatments, particularly at cooler temperatures (Figure 2a). However, generally, the presence of other species caused declines in egg‐to‐adult viability (competition). While temperature rarely changed the direction of the species interaction, we did find evidence that competition tended to be larger at cooler temperatures between 16 and 23°C than at 28°C; this was particularly true for tropical species. In line with this result, we found species interactions played an important role in shaping the cool‐range limit of tropical *Drosophila* species, such that species that were more sensitive to the presence of an interacting species were more likely to have a “warmer” cool‐range limit. This result is in line with a recent meta‐analysis that linked species interactions to both the cool and warm‐range limits (Paquette & Hargreaves, 2021).

In some instances, species interactions are thought to be even more important than temperature in shaping cool‐range limits, with a recent study on birds showing that elevational range sizes are dictated more by species diversity than climate (Freeman et al., 2022). If species interactions are more important than currently assumed in dictating species range limits, climate change could drive ecological, competitive, and evolutionary release, allowing species to exploit/adapt to new environments (Alexander et al., 2022). Species interactions could also limit the capacity of species to shift their distribution to track optimal temperatures if species cannot expand their cool‐range limit (often predicted from species distributional models) because species interactions also dictate their range limits. More work is needed to untangle how climate and species interactions shape range limits (Alexander et al., 2022).

Egg‐to‐adult viability was not influenced by competition in *D. simulans* at any temperature, which was surprising given that studies have shown that *D. simulans* are often outcompeted by *D. melanogaster* (Davis et al., 1998; Tantawy & Soliman, 1967). However, egg‐to‐adult viability is just one trait that may be involved in a species' competitive ability and other traits, such as fecundity and development time, are likely to contribute to the overall fitness of a species. Alternatively, species that remain viable under competition may do so at a cost to other traits such as body size or fecundity. This could translate into fitness consequences across generations. These types of trade‐offs and carry‐over effects, if present, could be crucial in understanding competitive ability and outcomes between species (Ezeakacha & Yee, 2019; Moore & Martin, 2019). There is some evidence from *Drosophila* experiments that competitive interactions can have lasting effects on body size, fecundity, and development long after competitors are removed (Grainger et al., 2021). Further work is needed to determine how commonly species interactions result in trade‐offs and carry‐over effects that dictate competitive outcomes.

We modeled the TPCs using the quadratics of temperature and the zero‐boundary enforced by a binomial regression. This approach is one of many possible ways to model TPCs and was chosen as it also allowed us to account for the overdispersion common to egg‐to‐adult viability and random effects of day when testing for the impact of competition on the TPC. We did not control for intraspecific interactions but rather controlled for absolute density. In some cases, intraspecific interactions have larger effects on fitness than interspecific interactions (Wallace, 1974). However, our preliminary study focusing explicitly on intraspecific interactions found that increasing density from 10 to 160 eggs had little effect on egg‐to‐adult viability in most species (Appendix S1: Figure S1). This suggests that the level of intraspecific interactions had only a small impact on egg‐to‐adult viability, and changes in intraspecific densities between competition and control vials were unlikely to affect the estimates from the TPCs. Based on the intraspecific competition results (Appendix S1: Figure S1), we also think it is unlikely that differences in absolute densities between the two‐way (80 eggs) and three‐way competition treatments (78 eggs) affected our results, but we cannot rule it out. Furthermore, we considered a single population for each species, so we cannot exclude the possibility that competitive ability may differ across populations. However, a study in *D. melanogaster* found no evidence for population variation in competitive ability (larval survival) when comparing a high‐ and low‐latitude population collected in Australia (James & Partridge, 1998). Nonetheless, a more extensive study of population variation in competitive ability would be valuable to determine whether populations differ in their competitive ability across environmental gradients.

As the climate changes, species will face increasing temperatures, as well as changes in community composition and how species interact. Here, we showed that the presence of other species tended to negatively impact egg‐to‐adult viability (competition). While we found species interactions changed the shape of the TPC in some species, we did not see changes in *T*_OPT_, questioning the predictive power of metrics like *T*_OPT_ for understanding climate change resilience within species. We found species interactions to have larger effects on egg‐to‐adult viability at cooler temperatures, particularly for tropical species. As such, tropical species tended to be more sensitive to the presence of other species at cooler temperatures, with species interactions likely to shape cool‐range limits. These results suggest that tropical species are more likely to be impacted by changing interactions under climate change. As the climate changes, species are expected to shift their distribution into higher latitude environments, but if species interactions are also crucial in shaping cool‐range limits, as we have shown here, the degree to which species can indeed shift to high‐latitude environments could be overestimated if species interactions limit distributional shifts.

## AUTHOR CONTRIBUTIONS

Vanessa Kellermann, Belinda van Heerwaarden, Tarmo Ketola, and Carla M. Sgrò conceptualized and designed the experiment. Vanessa Kellermann, Belinda van Heerwaarden, Tarmo Ketola, and Antti Miettinen conducted the experiments. Vanessa Kellermann and Mads F. Schou analyzed the data. Vanessa Kellermann, Mads F. Schou, and Belinda van Heerwaarden led the writing of the manuscript, and all authors contributed critically to drafts and gave final approval for publication.

## CONFLICT OF INTEREST STATEMENT

The authors declare no conflicts of interest.

## Supporting information


Appendix S1.

## Data Availability

Data and code (Kellermann et al., 2026) are available in Zenodo at https://doi.org/10.5281/zenodo.18822355.
