# Supplementary material for: Species interactions shape the thermal performance curve and distribution of tropical Drosophila species
Source: Ecology. 2026 Jul 8;107(7):e70451. doi: 10.1002/ecy.70451 (PMC13345751; doi:10.1002/ecy.70451)
Supplement: Supplementary file 1 — Appendix S1. [file ECY-107-e70451-s001.pdf]

Appendix S1

Species interactions shape the thermal performance curve and distribution of tropical *Drosophila* species

Vanessa Kellermann, Antti Miettinen, Carla M. Sgrò, Tarmo Ketola, Mads F. Schou and Belinda van Heerwaarden

Ecology

Supplementary methods and results

Intra-specific effects on egg-to-adult viability

To determine the extent to which intra-specific interactions might alter egg-to-adult viability, and whether intra-specific effects could be temperature-specific, we set up single-species cultures with different numbers of eggs added to each vial. We chose five different intraspecific densities: 10, 20, 40, 80 and 160 and estimated how viability changed across these densities at two different constant temperatures. For the sub-tropical species, we chose 23 and 28 ˚C, while for the tropical species, we chose 23 and 26 ˚C, as we were concerned that 28 ˚C would be stressful for this group of species (Kellermann and Sgro 2018). Prior to the experiment, 50-100 females were placed into six laying pots (at 25 ˚C) containing standard fly food (see above) spread with live yeast to stimulate oviposition. Eggs from these females were placed into vials containing 5 ml of standard fly food in the densities described above and placed into the different temperatures. The egg-picking occurred across two-days with laying plates replaced twice a day. We attempted to pick all treatments equally across both days but this was not always possible. For each species, density and temperature, we had 9-18 replicate vials with the majority of treatments having 12 replicate vials. Upon emergence, adults were counted, providing us with an estimate of egg-to-adult viability.

To examine whether varying the density of individuals of the same species within a vial influenced egg-to-adult viability we performed a linear model using the car package in R. As egg-to-adult viability is measured as the proportion of viable offspring we used an arcsin arcsin square root transformation to satisfy assumptions of normality. We included density

(continuous), temperature (continuous) and block (factor with two levels) as fixed effects, where block was the day of egg-picking.

Results: Intra-specific effects on viability

Generally, increasing levels of intra-specific interactions had only small effects on egg-to-adult viability. For the species where increasing levels of intra-specific interactions reduced egg-to-adult viability, this was only observed at a single temperature. In the sub-tropical species *D. serrata,* egg-to-adult viability decreased by 16% when developmental density increased (difference between the 10-160 density treatment) (23 ˚C: $F=6.31_{(1,62)}$, $P=0.01$) (Figure S1). The only other sub-tropical species where an effect of increasing density was observed was *D. simulans*, where viability decreased by 7% between the lowest and highest density (28 ˚C: $F=4.37_{(1,62)}$, $P=0.04$). Of the tropical species, only viability in *D. rubida* was impacted by increasing developmental density, declining by ~37% between the 10 and 160 density at 23 ˚C, (23 ˚C: $F=13.61_{(1,67)}$, $P<0.001$). Thus, impacts of density on egg-to-adult viability depended on temperature for only three species *D. serrata, D. simulans* and *D. rubida*.

Confirmation of species identification for D. melanogaster and D. simulans at two temperatures

Previous experience initiating field lines of *D. melanogaster* and *D. simulans* (where the species collected are often a mix of both species) collected post-summer across the Australian east coast cline meant VK was confident in her ability to identify the two species based on abdominal banding patterns of the females (males are easily distinguished by their genitalia) across different temperatures. Two temperatures were chosen, 23 °C and 30 °C, to further quantify the accuracy of the species identification. We chose 30 °C as warmer temperatures cause banding patterns to become lighter, making species identification more challenging, and 23 °C to confirm species identification at a benign temperature. For each of these temperatures, competition vials were initiated in the same way as the original experiment, i.e. flies of each species were placed into species-specific laying pots, and 40 eggs of each species were placed into one vial. For each temperature, five vials were initiated. On eclosion, emerging flies were collected into a vial and allowed to mate for 48hrs. Flies were then knocked out with CO_2 anesthesia, and from each vial, four females from each species were chosen and placed individually in a vial and placed at 25 °C (20 vials of each species for each

temperature). Flies were left until evidence of larvae in the food and then the female discarded. On emergence of the F1 generation, the identification of each species was confirmed by examining the male genitalia.

Results: species identification at 23 and 30 °C

From flies emerging from 23 °C, all 20 vials of *D. melanogaster* and *D. simulans* were identified correctly. From flies emerging from 30 °C, all flies emerging from the *D. melanogaster* vials were also identified correctly; however, 3 of 20 vials of the *D. simulans* vials were incorrectly identified as *D. simulans* when in fact they were *D. melanogaster.* The overall accuracy of species identification at temperatures ≥ 28 °C (given we did not test 28 °C we will assume our accuracy is comparable to 30 °C) was 92.50%.

Supplementary Figures

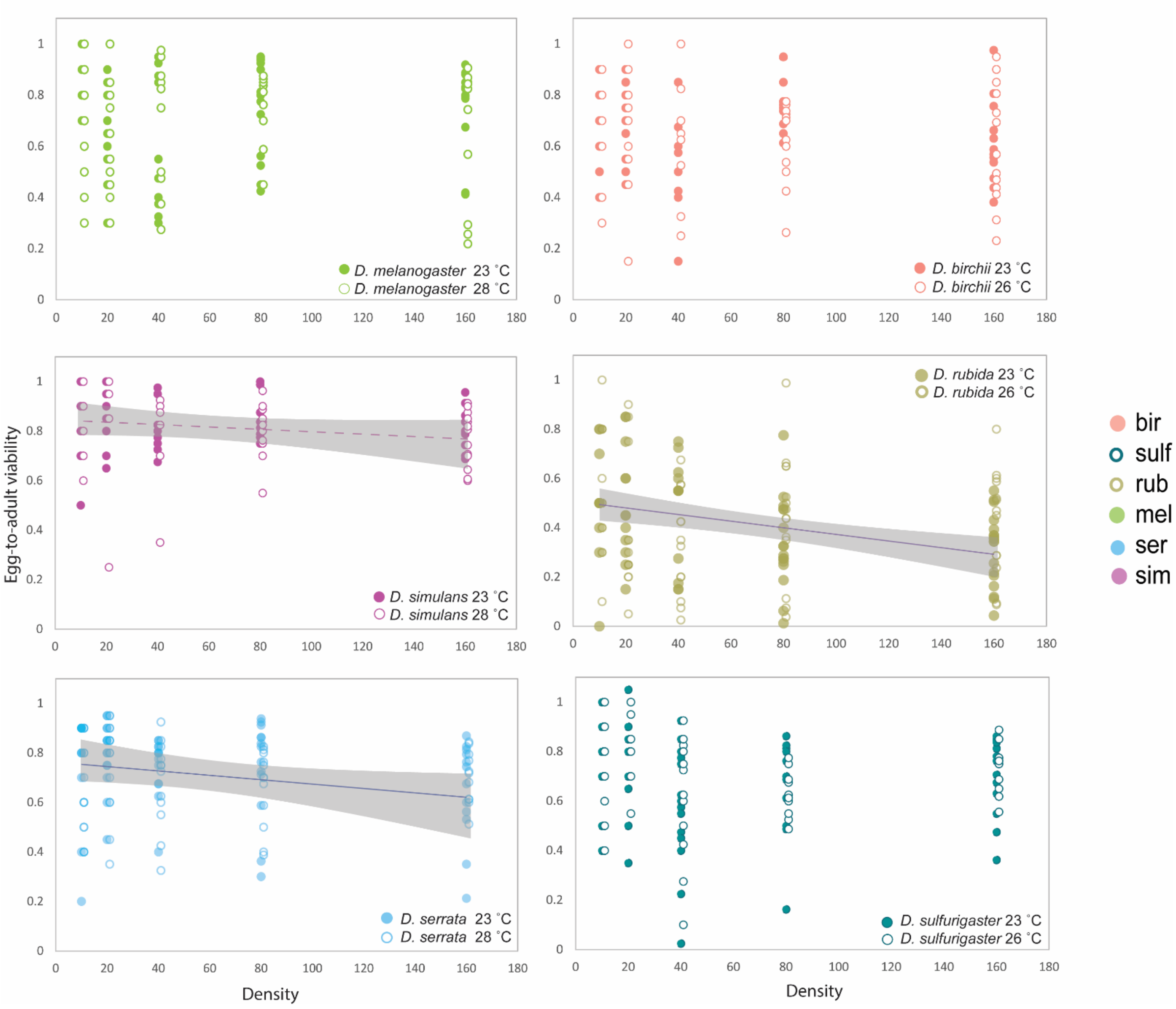

Figure S1. The effect of intra-specific interactions on egg-to-adult viability for six Drosophila species reared in single-species culture. The relationship between density and egg-to-adult viability at 23 and 28 ° C. Regression lines are given for significant density effects, with solid lines representing 23 °C and dashed lines representing 28 °C culture treatments. The line of best fit represents the respective 95% CoI around the mean prediction.

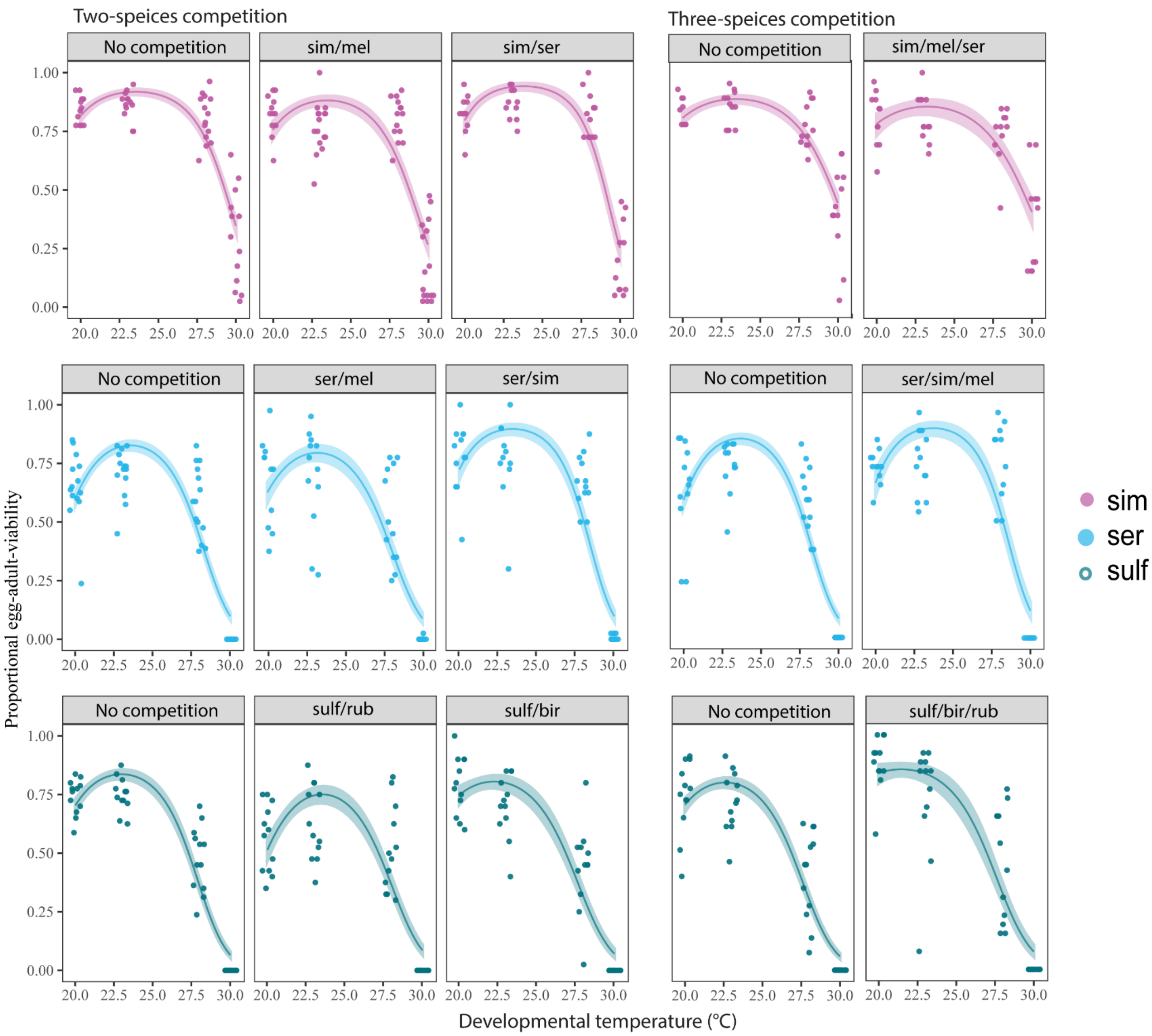

Figure S2. Thermal performance curves no 16 °C developmental temperature for *D. simulans*, *D. serrata*, and *D. sulfurigaster* with raw data from single-, two-, and three-species culture treatments. The left three panels show two species cultures, and the right two panels show three species cultures. 16 °C developmental temperature was removed to assess the fit of the curve.

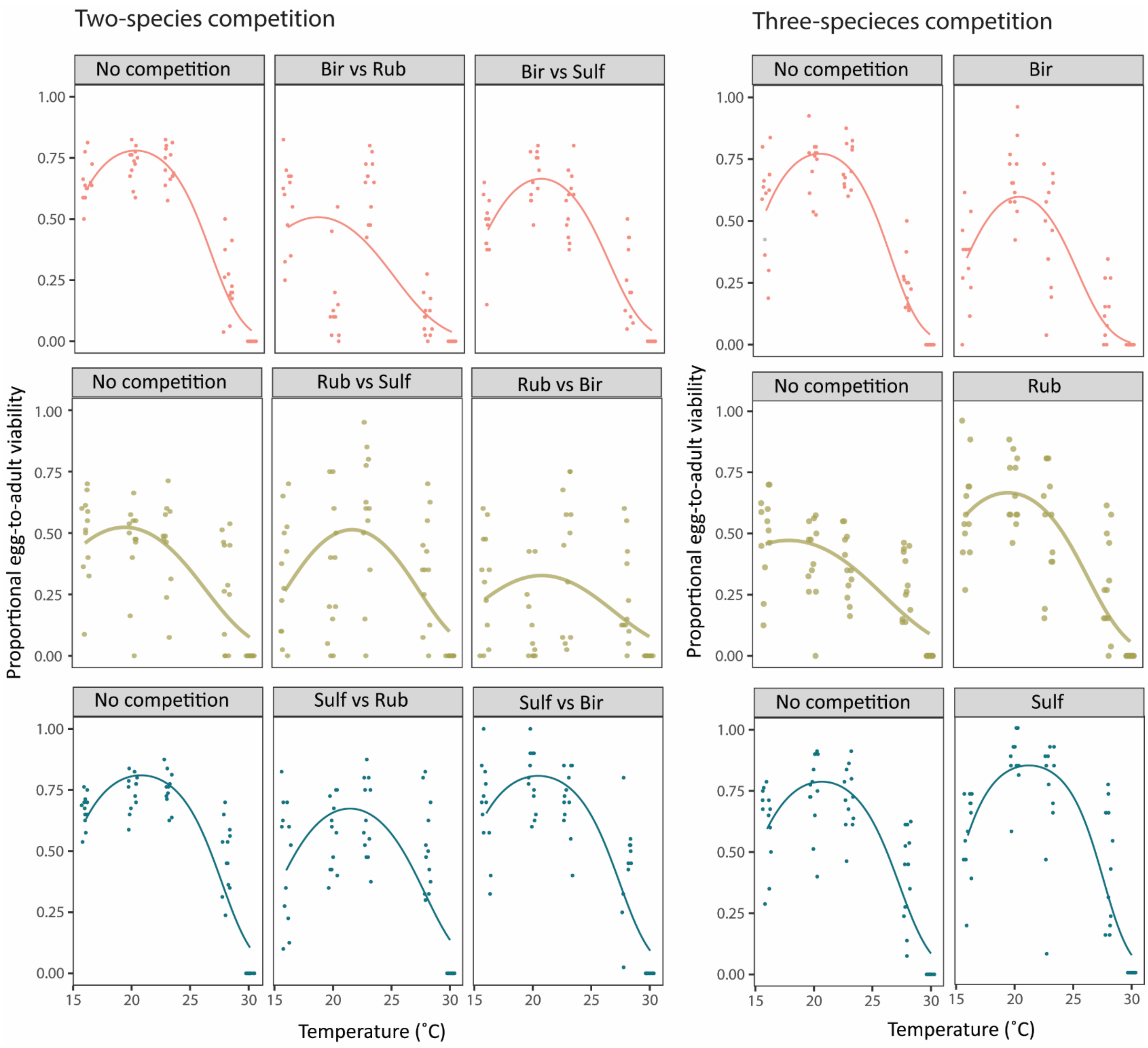

Figure S3. Thermal performance curves for *D. birchii*, *D. sulfurigaster*, and *D. rubida* with raw data from single-, two-, and three-species culture treatments. The left three panels show two species cultures, and the right two panels show three species cultures.

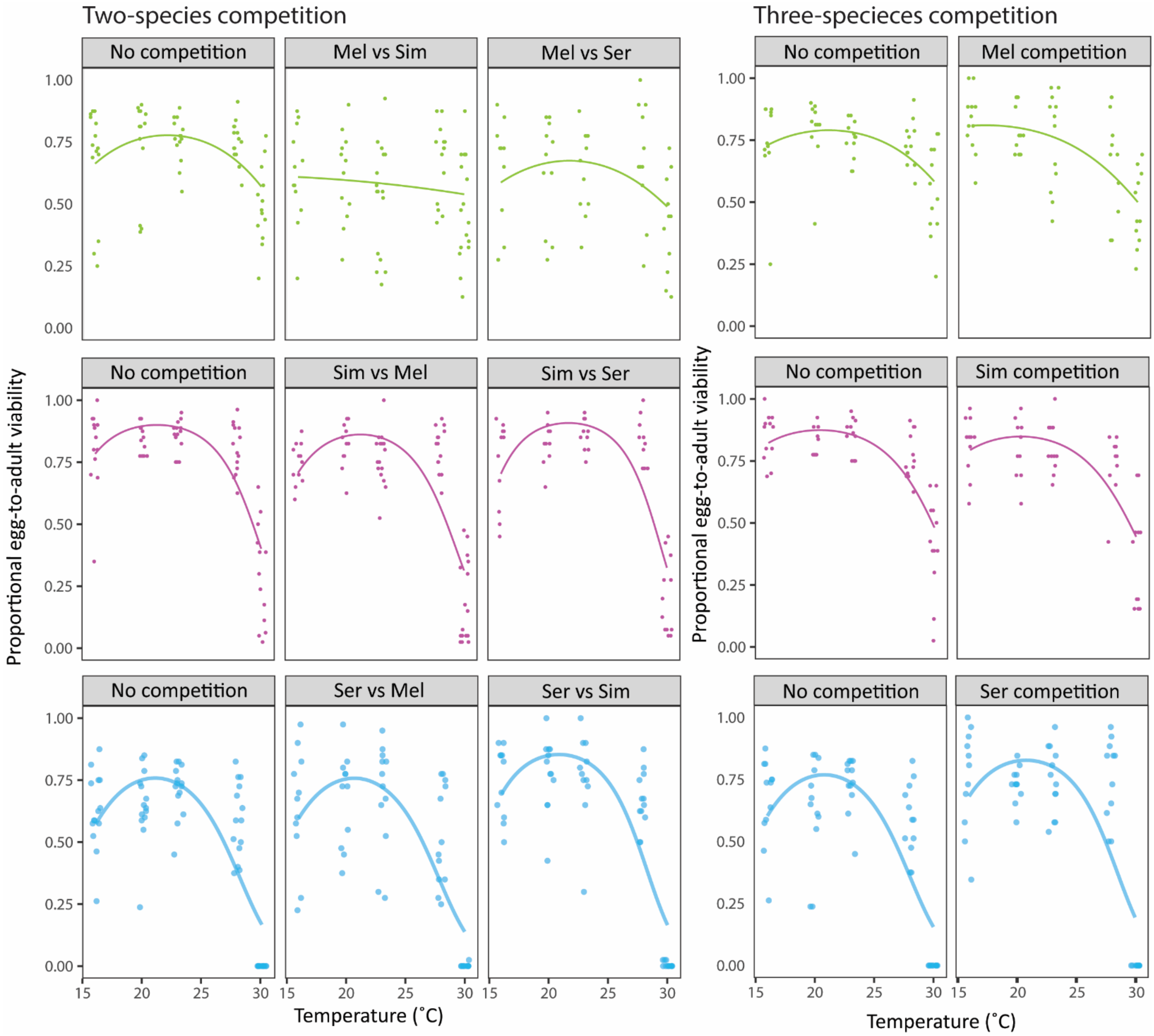

Figure S4. Thermal performance curves for *D. melanogaster*, *D. simulans*, and *D. serrata* with raw data from single-, two-, and three-species culture treatments. The left three panels show two species cultures, and the right two panels show three species cultures.

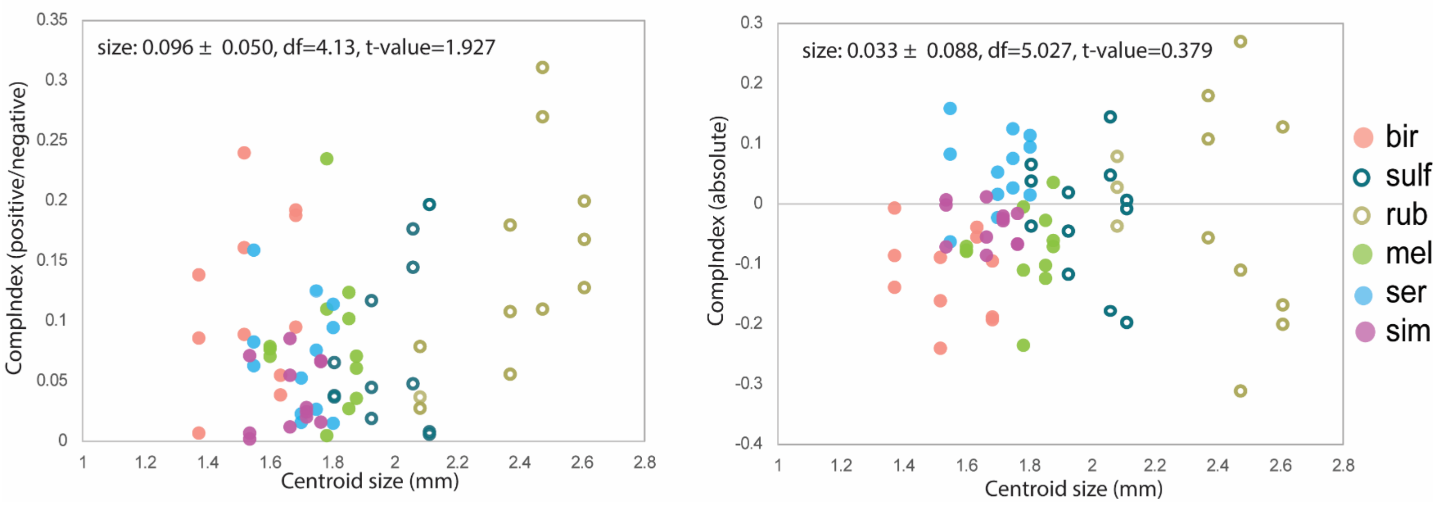

Figure S5. The relationship between wing size (a proxy for body size) and competitive outcomes (measured as the CompIndex). Fixed effect estimates for the relationship between CompIndex and size (β) are shown with their standard errors (SE), approximate degrees of freedom (df), and t-values. Significant effects ($p < 0.05$) are indicated in bold. The CompIndex for the bir/rub 20°C treatment culture was an outlier and removed from the model to ensure it was not influencing the results.

Table S1. Information on the species examined in the current study, including: collection time, site, GPS co-ordinates of collection sites in Australia and the number of generations in the lab prior to the experiments.

	**Collection time**	**Collection sites**	**GPS (latitude, longitude)**	**Generations in lab**
**Tropical**				
D. birchii	March 2014	Finch Hatton	-21.07, 148.63	17
D. sulfurigaster	March 2014	Finch Hatton	-21.07, 148.63	15
D. rubida	March 2013	Cairns	-16.86, 145.67	24
**Sub-tropical**				
D. melanogaster	April 2014	Melbourne	-37.81, 145.02	20
D. serrata	March 2012	Yamba	-29.43, 153.37	36
D. simulans	March 2014	Melbourne	-37.81, 145.02	19

Table S2: Comparison of two-way and three-way controls from the TPC analyses

We estimated the thermal performance curve (TPC) of the focal species on its own ("2" as control for 2-way species interactions and "3" as control for the 3-way species interaction). Here, we compare the TPC of these two controls within each focal species. These contrasts were performed on the same models as shown in Tables S3-S11.

Type	Parameter	Estimate (CI)	pMCMC
**Contrasts: Control(2) vs Control (3)**	Mel(2) vs Mel(3)	-0.06 (-1.03,0.93)	0.896
	Mel(2):temperature_z vs Mel(3):temperature_z	0.05 (-0.22,0.34)	0.730
	Mel(2):temperature_z2 vs Mel(3):temperature_z2	-0.12 (-0.5,0.27)	0.526
	Ser(2) vs Ser(3)	-0.05 (-0.94,0.97)	0.921
	Ser(2):temperature_z vs Ser(3):temperature_z	0.14 (-0.5,0.72)	0.630
	Ser(2):temperature_z2 vs Ser(3):temperature_z2	0.04 (-0.7,0.73)	0.919
	Sim(2) vs Sim(3)	0.23 (-0.69,1.15)	0.591
	Sim(2):temperature_z vs Sim(3):temperature_z	-0.05 (-0.37,0.26)	0.785
	Sim(2):temperature_z2 vs Sim(3):temperature_z2	-0.29 (-0.71,0.14)	0.186
	sulf2 vs sulf3	0.1 (-1.14,1.35)	0.834
	sulf2:temperature_z vs sulf3:temperature_z	0.09 (-0.41,0.57)	0.734
	sulf2:temperature_z2 vs sulf3:temperature_z2	0.07 (-0.58,0.64)	0.802
	rub2 vs rub3	0.06 (-1.54,1.89)	0.935
	rub2:temperature_z vs rub3:temperature_z	-0.21 (-0.87,0.47)	0.595
	rub2:temperature_z2 vs rub3:temperature_z2	-0.12 (-0.94,0.73)	0.790
	bir2 vs bir3	-0.12 (-0.91,0.54)	0.724
	bir2:temperature_z vs bir3:temperature_z	-0.11 (-0.54,0.35)	0.605
	bir2:temperature_z2 vs bir3:temperature_z2	0.15 (-0.37,0.67)	0.562

Estimate: Posterior mean is used for fixed effects and posterior mode is used for random effects

Table S3. Thermal performance curve analyses of temperate species *D. serrata*, excluding the 16 °C developmental temperature. We estimated the thermal performance curve (TPC) of each treatment and compared their linear and quadratic temperature effects. Treatments included 1) the focal species on its own ("2" as control for 2-way species interactions and "3" as control for the 3-way species interaction), 2) two 2-way species interactions and 3) one 3-way species interaction.

Type	Parameter	Estimate (CI)	pMCMC
**Fixed effects**	Ser(2)	1.65 (0.95,2.39)	**0**
**Fixed effects**	Ser/Sim	2.11 (1.28,2.99)	**0**
**Fixed effects**	Ser/Mel	1.43 (0.63,2.26)	**0.002**
**Fixed effects**	Ser(3)	1.81 (1,2.64)	**0**
**Fixed effects**	Ser/Mel/Sim	2.17 (1.36,3.14)	**0**
**Fixed effects**	Ser(2):temperature_z	-2.15 (-2.66,-1.72)	**0**
**Fixed effects**	Ser/Sim:temperature_z	-2.2 (-2.65,-1.66)	**0**
**Fixed effects**	Ser/Mel:temperature_z	-2.09 (-2.61,-1.57)	**0**
**Fixed effects**	Ser(3):temperature_z	-2.28 (-2.79,-1.82)	**0**
**Fixed effects**	Ser/Mel/Sim:temperature_z	-1.9 (-2.43,-1.38)	**0**
**Fixed effects**	Ser(2):temperature_z2	-2.48 (-3.19,-1.78)	**0**
**Fixed effects**	Ser/Sim:temperature_z2	-2.29 (-3.1,-1.54)	**0**
**Fixed effects**	Ser/Mel:temperature_z2	-2.14 (-2.89,-1.38)	**0**
**Fixed effects**	Ser(3):temperature_z2	-2.68 (-3.45,-2)	**0**
**Fixed effects**	Ser/Mel/Sim:temperature_z2	-2.39 (-3.24,-1.68)	**0**
**Random effect var.**	Day	0.009 (0,0.111)	-
**Random effect var.**	Residuals	1.817 (1.459,2.455)	-
**Contrasts: control vs 2-way**	Ser(2) vs Ser/Mel	-0.84 (-1.9,0.23)	0.131
**Contrasts: control vs 2-way**	Ser(2) vs Ser/Sim	-0.3 (-1.37,0.85)	0.602

Type	Parameter	Estimate (CI)	pMCMC
**Contrasts: control vs 2-way**	Ser(2):temperature_z vs Ser/Mel:temperature_z	-0.06 (-0.76,0.62)	0.885
**Contrasts: control vs 2-way**	Ser(2):temperature_z vs Ser/Sim:temperature_z	0.05 (-0.67,0.67)	0.847
**Contrasts: control vs 2-way**	Ser(2):temperature_z2 vs Ser/Mel:temperature_z2	-0.35 (-1.43,0.66)	0.504
**Contrasts: control vs 2-way**	Ser(2):temperature_z2 vs Ser/Sim:temperature_z2	-0.19 (-1.2,0.92)	0.728
**Contrasts: 2-way vs 2-way**	Ser/Sim vs Ser/Mel	0.68 (-0.45,1.89)	0.244
**Contrasts: 2-way vs 2-way**	Ser/Sim:temperature_z vs Ser/Mel:temperature_z	-0.11 (-0.82,0.6)	0.766
**Contrasts: 2-way vs 2-way**	Ser/Sim:temperature_z2 vs Ser/Mel:temperature_z2	-0.17 (-1.21,0.91)	0.773
**Contrasts: control vs 3-way**	Ser(3):temperature_z vs Ser/Mel/Sim:temperature_z	-0.37 (-1.06,0.41)	0.295
**Contrasts: control vs 3-way**	Ser(3):temperature_z2 vs Ser/Mel/Sim:temperature_z2	-0.29 (-1.33,0.79)	0.591
**Contrasts: 2-way vs 3-way**	Linear: (control vs 3-way) vs (control vs Ser/Mel)	-0.3 (-1.33,0.64)	0.541
**Contrasts: 2-way vs 3-way**	Linear: (control vs 3-way) vs (control vs Ser/Sim)	-0.44 (-1.4,0.51)	0.393
**Contrasts: 2-way vs 3-way**	Qudratic: (control vs 3-way) vs (control vs Ser/Mel)	0.05 (-1.46,1.47)	0.939
**Contrasts: 2-way vs 3-way**	Qudratic: (control vs 3-way) vs (control vs Ser/Sim)	-0.09 (-1.5,1.37)	0.913

Table S4. Thermal performance curve analyses of temperate species *D. simulans,* excluding the 16 °C developmental temperature, to test the robustness of our results. We estimated the thermal performance curve (TPC) of each treatment and compared their linear and quadratic temperature effects. Treatments included 1) the focal species on its own ("2" as control for 2-way species interactions and "3" as control for the 3-way species interaction), 2) two 2-way species interactions and 3) one 3-way species interaction.

Type	Parameter	Estimate (CI)	pMCMC
**Fixed effects**	Sim(2)	2.14 (1.56,2.76)	0
**Fixed effects**	Mel/Sim	1.72 (1.09,2.32)	0
**Fixed effects**	Ser/Sim	2.48 (1.79,3.14)	0
**Fixed effects**	Sim(3)	1.88 (1.14,2.56)	0.003
**Fixed effects**	Sim/Mel/Ser	1.55 (0.74,2.25)	0.003
**Fixed effects**	Sim(2):temperature_z	-1.05 (-1.28,-0.81)	0
**Fixed effects**	Sim/Mel:temperature_z	-1.13 (-1.36,-0.88)	0
**Fixed effects**	Sim/Ser:temperature_z	-1.27 (-1.55,-1.01)	0
**Fixed effects**	Sim(3):temperature_z	-0.84 (-1.08,-0.58)	0
**Fixed effects**	Sim/Mel/Ser:temperature_z	-0.86 (-1.13,-0.58)	0
**Fixed effects**	Sim(2):temperature_z2	-1.12 (-1.5,-0.72)	0
**Fixed effects**	Sim/Mel:temperature_z2	-1.1 (-1.51,-0.71)	0
**Fixed effects**	Sim/Ser:temperature_z2	-1.51 (-1.97,-1.09)	0
**Fixed effects**	Sim(3):temperature_z2	-0.86 (-1.26,-0.48)	0
**Fixed effects**	Sim/Mel/Ser:temperature_z2	-0.62 (-1.1,-0.12)	0.016
**Random effect var.**	Day	0.041 (0.001,0.55)	-
**Random effect var.**	Residuals	0.629 (0.5,0.819)	-
**Contrasts: control vs 2-way**	Sim(2) vs Sim/Mel	0.09 (-0.53,0.82)	0.784
**Contrasts: control vs 2-way**	Sim(2) vs Sim/Ser	0.3 (-0.42,0.94)	0.411

Type	Parameter	Estimate (CI)	pMCMC
**Contrasts: control vs 2-way**	Sim(2):temperature_z vs Sim/Mel:temperature_z	0.08 (-0.25,0.41)	0.644
**Contrasts: control vs 2-way**	Sim(2):temperature_z vs Sim/Ser:temperature_z	0.22 (-0.14,0.57)	0.225
**Contrasts: control vs 2-way**	Sim(2):temperature_z2 vs Sim/Mel:temperature_z2	-0.01 (-0.62,0.53)	0.968
**Contrasts: control vs 2-way**	Sim(2):temperature_z2 vs Sim/Ser:temperature_z2	0.39 (-0.16,0.99)	0.188
**Contrasts: 2-way vs 2-way**	Sim/Ser vs Sim/Mel	0.77 (0.07,1.46)	**0.03**
**Contrasts: 2-way vs 2-way**	Sim/Ser:temperature_z vs Sim/Mel:temperature_z	-0.14 (-0.49,0.22)	0.451
**Contrasts: 2-way vs 2-way**	Sim/Ser:temperature_z2 vs Sim/Mel:temperature_z2	-0.42 (-1.02,0.18)	0.19
**Contrasts: control vs 3-way**	Sim(3):temperature_z vs Sim/Mel/Ser:temperature_z	0.02 (-0.36,0.38)	0.908
**Contrasts: control vs 3-way**	Sim(3):temperature_z2 vs Sim/Mel/Ser:temperature_z2	-0.23 (-0.87,0.38)	0.473
**Contrasts: 2-way vs 3-way**	Linear: (control vs 3-way) vs (control vs Sim/Mel)	-0.06 (-0.58,0.44)	0.809
**Contrasts: 2-way vs 3-way**	Linear: (control vs 3-way) vs (control vs Sim/Ser)	-0.19 (-0.7,0.34)	0.453
**Contrasts: 2-way vs 3-way**	Qudratic: (control vs 3-way) vs (control vs Sim/Mel)	-0.22 (-1.06,0.62)	0.614
**Contrasts: 2-way vs 3-way**	Qudratic: (control vs 3-way) vs (control vs Sim/Ser)	-0.64 (-1.45,0.21)	0.165

Table S5. Thermal performance curve analyses of temperate species *D. sulfurigaster,* excluding the 16°C developmental temperature, to test the robustness of our results. We estimated the thermal performance curve (TPC) of each treatment and compared their linear and quadratic temperature effects. Treatments included 1) the focal species on its own ("2" as control for 2-way species interactions and "3" as control for the 3-way species interaction), 2) two 2-way species interactions and 3) one 3-way species interaction.

**Type**	**Parameter**	**Estimate (CI)**	**pMCMC**
**Fixed effects**	Sulf(2)	1.47 (0.34,2.45)	0.017
**Fixed effects**	Sulf/Bir	1.02 (-0.05,2.01)	0.057
**Fixed effects**	Sulf/Rub	1.2 (0.16,2.26)	0.032
**Fixed effects**	Sulf(3)	1.18 (0.02,2.41)	0.051
**Fixed effects**	Sulf/Bir/Rub	1.06 (-0.27,2.2)	0.08
**Fixed effects**	Sulf(2):temperature_z	-2.48 (-2.96,-2.07)	0
**Fixed effects**	Sulf/Bir:temperature_z	-2.4 (-2.86,-1.96)	0
**Fixed effects**	Sulf/Rub:temperature_z	-1.89 (-2.32,-1.41)	0
**Fixed effects**	Sulf(3):temperature_z	-2.59 (-3.02,-2.12)	0
**Fixed effects**	Sulf/Bir/Rub:temperature_z	-2.45 (-2.94,-1.94)	0
**Fixed effects**	Sulf(2):temperature_z2	-2.33 (-2.98,-1.63)	0
**Fixed effects**	Sulf/Bir:temperature_z2	-1.73 (-2.39,-1.09)	0
**Fixed effects**	Sulf/Rub:temperature_z2	-2.19 (-2.85,-1.51)	0
**Fixed effects**	Sulf(3):temperature_z2	-2.17 (-2.87,-1.55)	0
**Fixed effects**	Sulf/Bir/Rub:temperature_z2	-1.29 (-2.07,-0.62)	0.001
**Random effect var.**	Day	0.097 (0,1.883)	-
**Random effect var.**	Residuals	1.291 (1.013,1.762)	-
**Contrasts: control vs 2-way**	Sulf(2) vs Sulf/Bir	-0.48 (-1.48,0.47)	0.313
**Contrasts: control vs 2-way**	Sulf(2) vs Sulf/Rub	0.82 (-0.06,1.78)	0.08

**Type**	**Parameter**	**Estimate (CI)**	**pMCMC**
**Contrasts: control vs 2-way**	Sulf(2):temperature_z vs Sulf/Bir:temperature_z	-0.09 (-0.7,0.58)	0.775
**Contrasts: control vs 2-way**	Sulf(2):temperature_z vs Sulf/Rub:temperature_z	-0.59 (-1.21,0)	0.052
**Contrasts: control vs 2-way**	Sulf(2):temperature_z2 vs Sulf/Bir:temperature_z2	-0.61 (-1.45,0.4)	0.209
**Contrasts: control vs 2-way**	Sulf(2):temperature_z2 vs Sulf/Rub:temperature_z2	-0.12 (-1.09,0.77)	0.771
**Contrasts: 2-way vs 2-way**	Sulf/Rub vs Sulf/Bir	0.17 (-0.78,1.13)	0.718
**Contrasts: 2-way vs 2-way**	Sulf/Rub:temperature_z vs Sulf/Bir:temperature_z	0.5 (-0.09,1.18)	0.113
**Contrasts: 2-way vs 2-way**	Sulf/Rub:temperature_z2 vs Sulf/Bir:temperature_z2	-0.47 (-1.33,0.52)	0.349
**Contrasts: control vs 3-way**	Sulf(3):temperature_z vs Sulf/Bir/Rub:temperature_z	-0.14 (-0.78,0.54)	0.694
**Contrasts: control vs 3-way**	Sulf(3):temperature_z2 vs Sulf/Bir/Rub:temperature_z2	0.15 (-0.47,0.79)	0.589
**Contrasts: 2-way vs 3-way**	Linear: (control vs 3-way) vs (control vs Sulf/Bir)	-0.08 (-0.9,0.52)	0.545
**Contrasts: 2-way vs 3-way**	Linear: (control vs 3-way) vs (control vs Sulf/Rub)	0.5 (-0.22,1.19)	0.249
**Contrasts: 2-way vs 3-way**	Qudratic: (control vs 3-way) vs (control vs Sulf/Bir)	0.37 (-0.61,1.16)	0.486
**Contrasts: 2-way vs 3-way**	Qudratic: (control vs 3-way) vs (control vs Sulf/Rub)	0.51 (-0.51,1.23)	0.393

Table S6. Thermal performance curve analyses of temperate species *D. melanogaster*. We estimated the thermal performance curve (TPC) of each treatment and compared their linear and quadratic temperature effects. Treatments included 1) the focal species on its own ("2" as control for 2-way species interactions and "3" as control for the 3-way species interaction), 2) two 2-way species interactions and 3) one 3-way species interaction.

Type	Parameter	Estimate (CI)	pMCMC
**Fixed effects**	Mel/Ser	0.78 (0.19,1.39)	**0.024**
**Fixed effects**	Mel (3 species control)	1.32 (0.51,2.03)	**0.006**
**Fixed effects**	Mel/Ser/Sim	1.3 (0.57,2.13)	**0.008**
**Fixed effects**	Mel (2):temperature_z	-0.28 (-0.46,-0.08)	**0.005**
**Fixed effects**	Mel/Sim:temperature_z	-0.1 (-0.32,0.08)	0.319
**Fixed effects**	Mel/Ser:temperature_z	-0.23 (-0.44,0.01)	**0.042**
**Fixed effects**	Mel(3):temperature_z	-0.33 (-0.53,-0.11)	**0.001**
**Fixed effects**	Mel/Ser/Sim:temperature_z	-0.66 (-0.9,-0.43)	**0**
**Fixed effects**	Mel(2):temperature_z2	-0.43 (-0.67,-0.17)	**0.002**
**Fixed effects**	Mel/Sim:temperature_z2	0.07 (-0.2,0.33)	0.605
**Fixed effects**	Mel/Ser:temperature_z2	-0.27 (-0.53,0.04)	0.081
**Fixed effects**	Mel(3):temperature_z2	-0.31 (-0.56,-0.01)	**0.03**
**Fixed effects**	Mel/Ser/Sim:temperature_z2	-0.22 (-0.52,0.09)	0.166
**Random effect var.**	Day	0.077 (0.011,0.725)	
**Random effect var.**	Residuals	0.561 (0.468,0.706)	
**Contrasts: control vs 2-way**	Mel(2) vs Mel/Ser	0.55 (0.04,1)	**0.048**
**Contrasts: control vs 2-way**	Mel(2) vs Mel/Sim	0.96 (0.57,1.43)	**0**
**Contrasts: control vs 2-way**	Mel(2):temperature_z vs Mel/Ser:temperature_z	-0.1 (-0.34,0.25)	0.743

**Type**	**Parameter**	**Estimate (CI)**	**pMCMC**
**Contrasts: control vs 2-way**	Mel(2):temperature_z vs Mel/Sim:temperature_z	-0.17 (-0.44,0.11)	0.236
**Contrasts: control vs 2-way**	Mel(2):temperature_z2 vs Ser/Mel:temperature_z2	-0.21 (-0.54,0.21)	0.406
**Contrasts: control vs 2-way**	Mel(2):temperature_z2 vs Mel/Sim:temperature_z2	-0.6 (-0.85,-0.12)	**0.008**
**Contrasts: 2-way vs 2-way**	Mel/Sim vs Mel/Ser	-0.53 (-0.98,-0.01)	**0.039**
**Contrasts: 2-way vs 2-way**	Mel/Sim:temperature_z vs Mel/Ser:temperature_z	0.12 (-0.17,0.44)	0.401
**Contrasts: 2-way vs 2-way**	Mel/Sim:temperature_z2 vs Mel/Ser:temperature_z2	0.31 (-0.04,0.71)	0.088
**Contrasts: control vs 3-way**	Mel(3):temperature_z vs Mel/Ser/Sim:temperature_z	0.3 (0.04,0.67)	**0.041**
**Contrasts: control vs 3-way**	Mel(3):temperature_z2 vs Mel/Ser/Sim:temperature_z2	-0.09 (-0.52,0.31)	0.687
**Contrasts: 2-way vs 3-way**	Linear: (control vs 3-way) vs (control vs Mel/Ser)	0.43 (-0.08,0.8)	0.093
**Contrasts: 2-way vs 3-way**	Linear: (control vs 3-way) vs (control vs Mel/Sim)	0.55 (0.08,0.93)	**0.017**
**Contrasts: 2-way vs 3-way**	Qudratic: (control vs 3-way) vs (control vs Mel/Ser	0.04 (-0.52,0.62)	0.782
**Contrasts: 2-way vs 3-way**	Qudratic: (control vs 3-way) vs (control vs Mel/Sim)	0.45 (-0.13,0.97)	0.16

Table S7. Thermal performance curve analyses of temperate species *D. serrata*. We estimated the thermal performance curve (TPC) of each treatment and compared their linear and quadratic temperature effects. Treatments included 1) the focal species on its own ("2" as control for 2-way species interactions and "3" as control for the 3-way species interaction), 2) two 2-way species interactions and 3) one 3-way species interaction.

Type	Parameter	Estimate (CI)	pMCMC
**Fixed effects**	Ser(2)	1.16 (0.55,1.76)	**0.001**
**Fixed effects**	Ser/Sim	1.78 (1.04,2.46)	**0**
**Fixed effects**	Ser/Mel	1.19 (0.51,1.92)	**0.002**
**Fixed effects**	Ser(3)	1.2 (0.46,1.91)	**0.005**
**Fixed effects**	Ser/Mel/Sim	1.54 (0.75,2.25)	**0.001**
**Fixed effects**	Ser(2):temperature_z	-1.63 (-2.06,-1.21)	**0**
**Fixed effects**	Ser/Sim:temperature_z	-1.73 (-2.13,-1.24)	**0**
**Fixed effects**	Ser/Mel:temperature_z	-1.67 (-2.13,-1.24)	**0**
**Fixed effects**	Ser(3):temperature_z	-1.78 (-2.27,-1.37)	**0**
**Fixed effects**	Ser/Mel/Sim:temperature_z	-1.6 (-2.08,-1.09)	**0**
**Fixed effects**	Ser(2):temperature_z2	-1.63 (-2.14,-1.16)	**0**
**Fixed effects**	Ser/Sim:temperature_z2	-1.66 (-2.24,-1.12)	**0**
**Fixed effects**	Ser/Mel:temperature_z2	-1.54 (-2.09,-0.97)	**0**
**Fixed effects**	Ser(3):temperature_z2	-1.67 (-2.24,-1.17)	**0**
**Fixed effects**	Ser/Mel/Sim:temperature_z2	-1.34 (-1.95,-0.77)	**0**
**Random effect var.**	Day	0.016 (0,0.218)	
**Random effect var.**	Residuals	2.239 (1.754,2.779)	
**Contrasts: control vs 2-way**	Ser(2) vs Ser/Mel	-0.14 (-0.93,0.87)	0.958
**Contrasts: control vs 2-way**	Ser(2) vs Ser/Sim	-0.61 (-1.47,0.28)	0.17
**Contrasts: control vs 2-way**	Ser(2):temperature_z vs Ser/Mel:temperature_z	0.02 (-0.5,0.71)	0.899
**Contrasts: control vs 2-way**	Ser(2):temperature_z vs Ser/Sim:temperature_z	0.09 (-0.56,0.71)	0.727
**Contrasts: control vs 2-way**	Ser(2):temperature_z2 vs Ser/Mel:temperature_z2	0 (-0.87,0.64)	0.829
**Contrasts: control vs 2-way**	Ser(2):temperature_z2 vs Ser/Sim:temperature_z2	0.11 (-0.77,0.74)	0.932

Type	Parameter	Estimate (CI)	pMCMC
**Contrasts: 2-way vs 2-way**	Ser/Sim vs Ser/Mel	0.78 (-0.41,1.49)	0.22
**Contrasts: 2-way vs 2-way**	Ser/Sim:temperature_z vs Ser/Mel:temperature_z	-0.23 (-0.67,0.55)	0.836
**Contrasts: 2-way vs 2-way**	Ser/Sim:temperature_z2 vs Ser/Mel:temperature_z2	-0.05 (-0.97,0.64)	0.739
**Contrasts: control vs 3-way**	Ser(3):temperature_z vs Ser/Mel/Sim:temperature_z	-0.04 (-0.82,0.52)	0.594
**Contrasts: control vs 3-way**	Ser(3):temperature_z2 vs Ser/Mel/Sim:temperature_z2	-0.32 (-1.06,0.46)	0.415
**Contrasts: 2-way vs 3-way**	Linear: (control vs 3-way) vs (control vs Ser/Mel)	-0.14 (-1.11,0.69)	0.628
**Contrasts: 2-way vs 3-way**	Linear: (control vs 3-way) vs (control vs Ser/Sim)	-0.43 (-1.2,0.66)	0.532
**Contrasts: 2-way vs 3-way**	Qudratic: (control vs 3-way) vs (control vs Ser/Mel)	-0.28 (-1.3,0.8)	0.653
**Contrasts: 2-way vs 3-way**	Qudratic: (control vs 3-way) vs (control vs Ser/Sim)	-0.33 (-1.34,0.76)	0.504

Table S8. Thermal performance curve analyses of temperate species *D. simulans*. We estimated the thermal performance curve (TPC) of each treatment and compared their linear and quadratic temperature effects. Treatments included 1) the focal species on its own (“2” as control for 2-way species interactions and “3” as control for the 3-way species interaction), 2) two 2-way species interactions and 3) one 3-way species interaction.

Type	Parameter	Estimate (CI)	pMCMC
**Fixed effects**	Sim)2)	1.96 (1.37,2.58)	**0**
**Fixed effects**	Mel/Sim	1.64 (1.11,2.26)	**0**
**Fixed effects**	Ser/Sim	2.12 (1.55,2.77)	**0**
**Fixed effects**	Sim(3)	1.73 (0.95,2.41)	**0.003**
**Fixed effects**	Sim/Mel/Ser	1.59 (0.85,2.36)	**0.004**
**Fixed effects**	Sim(2):temperature_z	-0.82 (-1.05,-0.6)	**0**
**Fixed effects**	Sim/Mel:temperature_z	-0.87 (-1.12,-0.64)	**0**

Type	Parameter	Estimate (CI)	pMCMC
**Fixed effects**	Sim/Ser:temperature_z	-0.87 (-1.13,-0.64)	**0**
**Fixed effects**	Sim(3):temperature_z	-0.77 (-1,-0.55)	**0**
**Fixed effects**	Sim/Mel/Ser:temperature_z	-0.81 (-1.07,-0.54)	**0**
**Fixed effects**	Sim(2):temperature_z2	-0.83 (-1.1,-0.53)	**0**
**Fixed effects**	Sim/Mel:temperature_z2	-0.92 (-1.2,-0.6)	**0**
**Fixed effects**	Sim/Ser:temperature_z2	-1.14 (-1.44,-0.8)	**0**
**Fixed effects**	Sim(3):temperature_z2	-0.54 (-0.84,-0.23)	**0**
**Fixed effects**	Sim/Mel/Ser:temperature_z2	-0.47 (-0.81,-0.13)	**0.005**
**Random effect var.**	Day	0.072 (0.013,0.636)	
**Random effect var.**	Residuals	0.704 (0.599,0.908)	
**Contrasts: control vs 2-way**	Sim(2) vs Sim/Mel	0.31 (-0.24,0.81)	0.215
**Contrasts: control vs 2-way**	Sim(2) vs Sim/Ser	-0.22 (-0.7,0.39)	0.562
**Contrasts: control vs 2-way**	Sim(2):temperature_z vs Sim/Mel:temperature_z	0.1 (-0.26,0.39)	0.734
**Contrasts: control vs 2-way**	Sim(2):temperature_z vs Sim/Ser:temperature_z	0.1 (-0.26,0.39)	0.734
**Contrasts: control vs 2-way**	Sim(2):temperature_z2 vs Sim/Mel:temperature_z2	0.12 (-0.33,0.49)	0.678
**Contrasts: control vs 2-way**	Sim(2):temperature_z2 vs Sim/Ser:temperature_z2	0.36 (-0.11,0.75)	0.155
**Contrasts: 2-way vs 2-way**	Sim/Ser vs Sim/Mel	0.57 (-0.07,1.02)	0.099
**Contrasts: 2-way vs 2-way**	Sim/Ser:temperature_z vs Sim/Mel:temperature_z	-0.01 (-0.33,0.35)	0.987
**Contrasts: 2-way vs 2-way**	Sim/Ser:temperature_z2 vs Sim/Mel:temperature_z2	-0.23 (-0.64,0.26)	0.342
**Contrasts: control vs 3-way**	Sim(3):temperature_z vs Sim/Mel/Ser:temperature_z	0.05 (-0.3,0.39)	0.822

Type	Parameter	Estimate (CI)	pMCMC
**Contrasts: control vs 3-way**	Sim(3):temperature_z2 vs Sim/Mel/Ser:temperature_z2	-0.09 (-0.51,0.4)	0.761
**Contrasts: 2-way vs 3-way**	Linear: (control vs 3-way) vs (control vs Sim/Mel)	-0.06 (-0.5,0.47)	0.945
**Contrasts: 2-way vs 3-way**	Linear: (control vs 3-way) vs (control vs Sim/Ser)	0.15 (-0.47,0.46)	0.973
**Contrasts: 2-way vs 3-way**	Qudratic: (control vs 3-way) vs (control vs Sim/Mel)	-0.22 (-0.76,0.48)	0.616
**Contrasts: 2-way vs 3-way**	Qudratic: (control vs 3-way) vs (control vs Sim/Ser)	-0.39 (-0.99,0.28)	0.24

Table S9. Thermal performance curve analyses of temperate species *D. sulfurigaster*. We estimated the thermal performance curve (TPC) of each treatment and compared their linear and quadratic temperature effects. Treatments included 1) the focal species on its own ("2" as control for 2-way species interactions and "3" as control for the 3-way species interaction), 2) two 2-way species interactions and 3) one 3-way species interaction.

Type	Parameter	Estimate (CI)	pMCMC
**Fixed effects**	Sulf(2)	1.31 (0.52,2.19)	**0.011**
**Fixed effects**	Sulf/Bir	1.29 (0.45,2.07)	**0.011**
**Fixed effects**	Sulf/Rub	0.79 (-0.04,1.69)	0.061
**Fixed effects**	Sulf(3)	1.2 (0.2,2.13)	**0.027**
**Fixed effects**	Sulf/Bir/Rub	1.84 (0.84,2.8)	**0.005**
**Fixed effects**	Sulf(2):temperature_z	-1.85 (-2.21,-1.52)	**0**
**Fixed effects**	Sulf/Bir:temperature_z	-1.88 (-2.24,-1.5)	**0**
**Fixed effects**	Sulf/Rub:temperature_z	-1.25 (-1.6,-0.88)	**0**
**Fixed effects**	Sulf(3):temperature_z	-1.93 (-2.27,-1.58)	**0**
**Fixed effects**	Sulf/Bir/Rub:temperature_z	-1.75 (-2.16,-1.36)	**0**
**Fixed effects**	Sulf(2):temperature_z2	-1.73 (-2.14,-1.28)	**0**

Type	Parameter	Estimate (CI)	pMCMC
**Fixed effects**	Sulf/Bir:temperature_z2	-1.58 (-2.03,-1.13)	**0**
**Fixed effects**	Sulf/Rub:temperature_z2	-1.51 (-1.96,-1.08)	**0**
**Fixed effects**	Sulf(3):temperature_z2	-1.81 (-2.24,-1.37)	**0**
**Fixed effects**	Sulf/Bir/Rub:temperature_z2	-1.98 (-2.46,-1.48)	**0**
**Random effect var.**	Day	0.096 (0,1.307)	
**Random effect var.**	Residuals	1.3 (1.018,1.665)	
**Contrasts: control vs 2-way**	Sulf(2) vs Sulf/Bir	-0.02 (-0.68,0.7)	0.963
**Contrasts: control vs 2-way**	Sulf(2) vs Sulf/Rub	0.45 (-0.2,1.2)	0.155
**Contrasts: control vs 2-way**	Sulf(2):temperature_z vs Sulf/Bir:temperature_z	0.01 (-0.46,0.54)	0.896
**Contrasts: control vs 2-way**	Sulf(2):temperature_z vs Sulf/Rub:temperature_z	-0.65 (-1.1,-0.12)	**0.018**
**Contrasts: control vs 2-way**	Sulf(2):temperature_z2 vs Sulf/Bir:temperature_z2	-0.11 (-0.74,0.48)	0.648
**Contrasts: control vs 2-way**	Sulf(2):temperature_z2 vs Sulf/Rub:temperature_z2	-0.19 (-0.85,0.38)	0.494
**Contrasts: 2-way vs 2-way**	Sulf/Rub vs Sulf/Bir	-0.59 (-1.26,0.15)	0.163
**Contrasts: 2-way vs 2-way**	Sulf/Rub:temperature_z vs Sulf/Bir:temperature_z	0.53 (0.13,1.15)	**0.017**
**Contrasts: 2-way vs 2-way**	Sulf/Rub:temperature_z2 vs Sulf/Bir:temperature_z2	0.15 (-0.56,0.67)	0.81
**Contrasts: control vs 3-way**	Sulf(3):temperature_z vs Sulf/Bir/Rub:temperature_z	-0.2 (-0.72,0.32)	0.489
**Contrasts: control vs 3-way**	Sulf(3):temperature_z2 vs Sulf/Bir/Rub:temperature_z2	0.15 (-0.47,0.79)	0.589
**Contrasts: 2-way vs 3-way**	Linear: (control vs 3-way) vs (control vs Sulf/Bir)	-0.08 (-0.9,0.52)	0.545

Type	Parameter	Estimate (CI)	pMCMC
**Contrasts: 2-way vs 3-way**	Linear: (control vs 3-way) vs (control vs Sulf/Rub)	0.5 (-0.22,1.19)	0.249
**Contrasts: 2-way vs 3-way**	Qudratic: (control vs 3-way) vs (control vs Sulf/Bir)	0.37 (-0.61,1.16)	0.486
**Contrasts: 2-way vs 3-way**	Qudratic: (control vs 3-way) vs (control vs Sulf/Rub)	0.51 (-0.51,1.23)	0.393

Table S10. Thermal performance curve analyses of temperate species *D. rubida*. We estimated the thermal performance curve (TPC) of each treatment and compared their linear and quadratic temperature effects. Treatments included 1) the focal species on its own ("2" as control for 2-way species interactions and "3" as control for the 3-way species interaction), 2) two 2-way species interactions and 3) one 3-way species interaction.

Type	Parameter	Estimate (CI)	pMCMC
**Fixed effects**	Rub(2)	-0.37 (-1.58,0.64)	0.46
**Fixed effects**	Rub/Bir	-1.28 (-2.48,-0.15)	**0.037**
**Fixed effects**	Rub/Sulf	-0.09 (-1.15,1.04)	0.849
**Fixed effects**	Rub(3)	-0.45 (-1.71,0.83)	0.417
**Fixed effects**	Rub/Bir/Sulf	0.37 (-0.83,1.77)	0.495
**Fixed effects**	Rub(2):temperature_z	-1.85 (-2.37,-1.36)	**0**
**Fixed effects**	Rub/Bir:temperature_z	-1.06 (-1.6,-0.57)	**0**
**Fixed effects**	Rub/Sulf:temperature_z	-1.21 (-1.72,-0.72)	**0**
**Fixed effects**	Rub(3):temperature_z	-1.65 (-2.13,-1.2)	**0**
**Fixed effects**	Rub/Bir/Sulf:temperature_z	-1.95 (-2.5,-1.46)	**0**
**Fixed effects**	Rub(2):temperature_z2	-1.31 (-1.9,-0.7)	**0**
**Fixed effects**	Rub/Bir:temperature_z2	-1.1 (-1.71,-0.47)	**0**
**Fixed effects**	Rub/Sulf:temperature_z2	-1.59 (-2.21,-0.98)	**0**
**Fixed effects**	Rub(3):temperature_z2	-1.19 (-1.81,-0.63)	**0**

Type	Parameter	Estimate (CI)	pMCMC
**Fixed effects**	Rub/Bir/Sulf:temperature_z2	-1.38 (-1.97,-0.78)	**0**
**Random effect var.**	Day	0.15 (0.001,1.993)	
**Random effect var.**	Residuals	2.49 (1.93,3.149)	
**Contrasts: control vs 2-way**	Rub(2) vs Rub/Bir	0.82 (-0.12,1.86)	0.068
**Contrasts: control vs 2-way**	Rub(2) vs Rub/Sulf	-0.39 (-1.2,0.71)	0.561
**Contrasts: control vs 2-way**	Rub(2):temperature_z vs Rub/Bir:temperature_z	-0.83 (-1.49,-0.04)	**0.033**
**Contrasts: control vs 2-way**	Rub(2):temperature_z vs Rub/Sulf:temperature_z	-0.61 (-1.34,0.05)	0.079
**Contrasts: control vs 2-way**	Rub(2):temperature_z2 vs Rub/Bir:temperature_z2	0.05 (-1.09,0.66)	0.645
**Contrasts: control vs 2-way**	Rub(2):temperature_z2 vs Rub/Sulf:temperature_z2	0.14 (-0.57,1.11)	0.505
**Contrasts: 2-way vs 2-way**	Rub/Sulf vs Rub/Bir	0.99 (0.25,2.21)	**0.013**
**Contrasts: 2-way vs 2-way**	Rub/Sulf:temperature_z vs Rub/Bir:temperature_z	-0.15 (-0.88,0.55)	0.693
**Contrasts: 2-way vs 2-way**	Rub/Sulf:temperature_z2 vs Rub/Bir:temperature_z2	-0.48 (-1.41,0.37)	0.256
**Contrasts: control vs 3-way**	Rub(3):temperature_z vs Rub/Bir/Sulf:temperature_z	0.22 (-0.43,0.94)	0.406
**Contrasts: control vs 3-way**	Rub(3):temperature_z2 vs Rub/Bir/Sulf:temperature_z2	0.21 (-0.66,1.05)	0.656
**Contrasts: 2-way vs 3-way**	Linear: (control vs 3-way) vs (control vs Rub/Bir)	1.01 (0.16,2.16)	**0.039**
**Contrasts: 2-way vs 3-way**	Linear: (control vs 3-way) vs (control vs Rub/Sulf)	1.09 (0,1.94)	0.06
**Contrasts: 2-way vs 3-way**	Qudratic: (control vs 3-way) vs (control vs Rub/Bir)	0.77 (-0.88,1.55)	0.522
**Contrasts: 2-way vs 3-way**	Qudratic: (control vs 3-way) vs (control vs Rub/Sulf)	-0.11 (-1.34,1.12)	0.899

Table S11. Thermal performance curve analyses of temperate species *D. birchii*. We estimated the thermal performance curve (TPC) of each treatment and compared their linear and quadratic temperature effects. Treatments included 1) the focal species on its own ("2" as control for 2-way species interactions and "3" as control for the 3-way species interaction), 2) two 2-way species interactions and 3) one 3-way species interaction.

**Type**	**Parameter**	**Estimate (CI)**	**pMCMC**
**Fixed effects**	Bir(2)	0.88 (0.34,1.39)	**0.003**
**Fixed effects**	Bir/Rub	-0.66 (-1.18,-0.1)	**0.023**
**Fixed effects**	Bir/Sulf	0.46 (-0.05,0.99)	0.085
**Fixed effects**	Bir(3)	1 (0.42,1.5)	**0.006**
**Fixed effects**	Bir/Rub/Sulf	0.03 (-0.58,0.55)	0.905
**Fixed effects**	Bir(2):temperature_z	-2.06 (-2.38,-1.76)	**0**
**Fixed effects**	Bir/Rub:temperature_z	-1.68 (-2,-1.32)	**0**
**Fixed effects**	Bir/Sulf:temperature_z	-1.67 (-2.01,-1.34)	**0**
**Fixed effects**	Bir(3):temperature_z	-1.95 (-2.27,-1.64)	**0**
**Fixed effects**	Bir/Rub/Sulf:temperature_z	-1.85 (-2.25,-1.45)	**0**
**Fixed effects**	Bir(2):temperature_z2	-1.67 (-2.05,-1.29)	**0**
**Fixed effects**	Bir/Rub:temperature_z2	-0.98 (-1.37,-0.61)	**0**
**Fixed effects**	Bir/Sulf:temperature_z2	-1.53 (-1.92,-1.16)	**0**
**Fixed effects**	Bir(3):temperature_z2	-1.81 (-2.17,-1.41)	**0**
**Fixed effects**	Bir/Rub/Sulf:temperature_z2	-1.66 (-2.11,-1.25)	**0**
**Random effect var.**	Day	0.003 (0,0.278)	
**Random effect var.**	Residuals	0.913 (0.726,1.151)	
**Contrasts: control vs 2-way**	Bir(2) vs Bir/Rub	1.59 (0.94,2.15)	**0**
**Contrasts: control vs 2-way**	Bir(2) vs Bir/Sulf	0.5 (-0.22,0.98)	0.167

**Type**	**Parameter**	**Estimate (CI)**	**pMCMC**
**Contrasts: control vs 2-way**	Bir(2):temperature_z vs Bir/Rub:temperature_z	-0.38 (-0.88,0.03)	0.103
**Contrasts: control vs 2-way**	Bir(2):temperature_z vs Bir/Sulf:temperature_z	-0.41 (-0.84,0.08)	0.106
**Contrasts: control vs 2-way**	Bir(2):temperature_z2 vs Bir/Rub:temperature_z2	-0.72 (-1.19,-0.13)	**0.013**
**Contrasts: control vs 2-way**	Bir(2):temperature_z2 vs Bir/Sulf:temperature_z2	-0.16 (-0.67,0.41)	0.615
**Contrasts: 2-way vs 2-way**	Bir/Sulf vs Bir/Rub	1.11 (0.49,1.69)	**0**
**Contrasts: 2-way vs 2-way**	Bir/Sulf:temperature_z vs Bir/Rub:temperature_z	0.08 (-0.48,0.46)	0.96
**Contrasts: 2-way vs 2-way**	Bir/Sulf:temperature_z2 vs Bir/Rub:temperature_z2	-0.68 (-1.1,0)	**0.041**
**Contrasts: control vs 3-way**	Bir(3):temperature_z vs Bir/Rub/Sulf:temperature_z	0 (-0.61,0.38)	0.711
**Contrasts: control vs 3-way**	Bir(3):temperature_z2 vs Bir/Rub/Sulf:temperature_z2	-0.04 (-0.73,0.41)	0.585
**Contrasts: 2-way vs 3-way**	Linear: (control vs 3-way) vs (control vs Bir/Rub)	0.24 (-0.38,0.95)	0.407
**Contrasts: 2-way vs 3-way**	Linear: (control vs 3-way) vs (control vs Bir/Sulf)	0.31 (-0.42,0.94)	0.407
**Contrasts: 2-way vs 3-way**	Qudratic: (control vs 3-way) vs (control vs Bir/Rub)	0.53 (-0.25,1.26)	0.174
**Contrasts: 2-way vs 3-way**	Qudratic: (control vs 3-way) vs (control vs Bir/Sulf)	-0.06 (-0.78,0.74)	0.964

Table S12. Maximum performance (P_{MAX}) for all focal species and their interacting species. Highlighted boxes indicate the controls (there are two sets of controls, one for each of the two- and three-way interactions). Significant contrasts between the controls and the two- and three-way interactions are highlighted in bold.

			**Interacting species**		
**Focal species**	**mel**	**sim**	**ser**	**control (3)**	**mel/sim/ser**
**mel**	0.79 (0.68, 0.87)	**0.56 (0.41, 0.70)**	**0.69 (0.56, 0.81)**	0.80 (0.65, 0.90)	0.86 (0.73, 0.93)
**sim**	0.86 (0.79, 0.92)	0.90 (0.83, 0.94)	0.91 (0.85, 0.95)	0.88 (0.79, 0.94)	0.87 (0.77, 0.94)
**ser**	0.84 (0.73,0.91)	0.90 (0.83,0.95)	0.83 (0.73, 0.90)	0.84 (0.73, 0.91)	0.88 (0.79, 0.94
	**sulf**	**bir**	**rub**	**control (3)**	**sulf/bir/rub**
**sulf**	0.86 (0.73, 0.94)	0.86 (0.76, 0.94)	0.86 (0.73, 0.94)	0.85 (0.68, 0.93)	0.9 (0.78, 0.96)
**bir**	**0.71 (0.60, 0.81)**	0.82 (0.73, 0.88)	**0.51 (0.4, 0.64)**	0.82 (0.74, 0.89)	**0.63 (0.5, 0.75)**
**rub**	0.53 (0.28, 0.77)	**0.27 (0.09, 0.5)**	0.57 (0.33, 0.81)	0.53 (0.28, 0.82)	**0.74 (0.46, 0.92)**

Table S13. Thermal optima (T_{OPT}) and the confidence intervals for all focal species and their interacting species. Highlighted boxes indicate the controls (there are two sets of controls, one for each of the two- and three-way interactions). Significant contrasts between the controls and the two- and three-way interactions are highlighted in bold.

	Interacting species				
**Focal species**	**mel**	**sim**	**ser**	**control (3)**	**mel/sim/ser**
**mel**	21.82 (19.95,23.24)	24.41 (-6.34,58.3)	21.45 (6.55,26.64)	20.82 (12.3,23.04)	16.84 (-52.83,70.19)
**sim**	21.07 (19.94,21.93)	20.99 (19.82,21.94)	21.55 (20.69,22.2)	19.88 (16.65,21.82)	19.18 (12.5,21.86)
**ser**	20.62 (19.3,21.69)	20.73 (19.62,21.71)	20.85 (19.88,21.64)	20.69 (19.48,21.48)	20.36 (18.59,21.69)
	**sulf**	**bir**	**rub**	**control (3)**	**sulf/bir/rub**
**sulf**	20.67 (19.9,21.35)	20.32 (19.35,21.17)	21.28 (20.43,21.97)	20.65 (19.89,21.35)	21.14 (20.48,21.71)
**bir**	20.6 (19.8,21.24)	20.24 (19.44,20.9)	19.01 (16.98,20.44)	20.64 (20.05,21.19)	20.54 (19.78,21.18)
**rub**	21.47 (20.36,22.32)	20.91 (18.65,22.26)	19.78 (17.65,21.2)	19.83 (17.48,21.43)	19.78 (17.72,21.18)

Table S14. The relationship between CompIndex (negative and positive), latitude and temperature from a linear mixed-effects model. Fixed effect estimates (β) are shown with their standard errors (SE), approximate degrees of freedom (df), and t-values. Significant effects ($p < 0.05$) are indicated in bold. Marginal and Conditional R^2 are shown portioning the variation attributed to the random and fixed effects in the model.

	Estimate	Df	t-values
Latitude	-0.13 ± 0.249	43.140	1.864
Temperature	0.021 ± 0.009	70.028	**2.109**
Latitude x Temperature	-0.001 ± 0.001	70.113	-1.97
R2m	0.045		
R2C	0.356		

Table S15. **The relationship between CompIndex (positive), latitude and temperature from a linear mixed-effects model.** Fixed effect estimates (β) are shown with their standard errors (SE), approximate degrees of freedom (df), and t-values. Significant effects ($p < 0.05$) are indicated in bold. Marginal and Conditional R^2 are shown portioning the variation attributed to the random and fixed effects in the model.

	Estimate	df	t-values
Latitude	-0.016 0.004	73.999	**-3.384**
Temperature	-0.021 0.007	70.749	**-2.899**
Latitude x Temperature	0.001 0.001	71.183	**2.688**
R2m	0.234		
R2C	0.243		

Reference

Kellermann, V. and C. M. Sgro (2018). "Evidence for lower plasticity in CTMAX at warmer developmental temperatures." Journal of Evolutionary Biology **31**(9): 1300-1312.
